# Two distinct types of the Indian Ocean Dipole

**DOI:** 10.1093/nsr/nwag519

**Published:** 2026-08-21

**Authors:** Jiayu Zheng, Chunzai Wang

**Affiliations:** Laboratory of Tropical Oceanography, South China Sea Institute of Oceanology, Chinese Academy of Sciences, Guangzhou 510301, China; Global Ocean and Climate Research Center, South China Sea Institute of Oceanology, Chinese Academy of Sciences, Guangzhou 510301, China; China-Sri Lanka Belt and Road Joint Laboratory on Tropical Oceanography, South China Sea Institute of Oceanology, Chinese Academy of Sciences, Guangzhou 510301, China; Laboratory of Tropical Oceanography, South China Sea Institute of Oceanology, Chinese Academy of Sciences, Guangzhou 510301, China; Global Ocean and Climate Research Center, South China Sea Institute of Oceanology, Chinese Academy of Sciences, Guangzhou 510301, China; China-Sri Lanka Belt and Road Joint Laboratory on Tropical Oceanography, South China Sea Institute of Oceanology, Chinese Academy of Sciences, Guangzhou 510301, China

**Keywords:** Indian Ocean Dipole, East African rainfall, climate modes

## Abstract

The Indian Ocean Dipole (IOD), traditionally defined by the sea surface temperature (SST) anomaly gradient between the tropical western and eastern Indian Ocean, is revealed here to comprise two distinct types. Using multiple systematic approaches, including standard deviation analysis and SST mode combinations, we identify a western–eastern type and a central–eastern type, distinguished by their SST anomaly patterns, seasonal evolution, and feedback mechanisms. The western–eastern type reaches its peak later in boreal autumn due to a strong but delayed wind–thermocline–SST feedback, whereas the central–eastern type peaks earlier, governed by a weaker and earlier feedback. Crucially, the positive western–eastern type—more strongly linked to enhanced East African rainfall than the traditional IOD—has intensified in recent decades, heightening the risk of rainfall-related disasters across East Africa. These findings refine the conceptual framework of the IOD and underscore the growing climate hazard posed by its changing characteristics.

## Teaser

The Indian Ocean Dipole exhibits two distinct spatiotemporal patterns, characterized by different ocean–atmosphere interactions and climate impacts. Distinguishing between these patterns provides new insights into how Indian Ocean variability shapes extreme rainfall over East Africa.

## INTRODUCTION

Sea surface temperature (SST) modes across the three tropical oceans exhibit significant interannual variability, profoundly shaping global climate and socioeconomic systems [1,2]. In the tropical Pacific, the classical eastern Pacific El Niño is the most extensively documented mode. Since the late 1990s, the central Pacific El Niño, or El Niño Modoki, characterized by a westward shift in SST warming, has become more frequent and affects climate in ways distinct from the classical type [3–6]. Similarly, in the tropical Atlantic, the Atlantic Niño underwent a notable interdecadal shift around the 2000s: the eastern Atlantic Niño weakened by ∼50%, while the central Atlantic Niño emerged as the dominant mode influencing ENSO [7].

In the tropical Indian Ocean, the Indian Ocean Dipole (IOD) is a prominent SST mode, characterized by the zonal gradient of SST anomalies between the western and eastern basin [8]. This classical mode, hereafter the traditional IOD (T-IOD), peaks during boreal autumn (September–November) and has profound impacts on rainfall in East Africa and India, as well as on droughts in East Asia and Australia [8–11]. Positive T-IOD events, in particular, often trigger severe flooding in East Africa, including Somalia and neighboring countries, causing widespread crop failures, damage to water infrastructure, and substantial economic losses [8].

To quantify this traditional dipole mode, the T-IOD index is defined as the SST anomaly difference between the western (50°E–70°E, 10°S–10°N) and eastern (90°E–110°E, 10°S–0°) tropical Indian Ocean boxes, designed to capture the dipole structure represented by the second empirical orthogonal function (EOF2) of SST anomalies [8]. Although widely used, EOF modes often lack direct physical interpretation and therefore require complementary validation [12,13]. Reproducing observed SST patterns typically requires multiple EOF modes, as a single mode generally provides insufficient explanatory power [7,14]. Consequently, the T-IOD alone may not fully capture the diversity and complexity of SST variability across the tropical Indian Ocean.

Since its identification, IOD events have been grouped into two or three categories according to their distinct characteristics [15–21]. From the perspective of seasonal evolution, they are classified as summer, autumn, and long-lasting events, with the latter developing in boreal summer and maturing in boreal autumn [15,16]. Based on eastern tropical Indian Ocean SST intensity, events are divided into strong and moderate [17–19]. Model simulations, such as those from the Pacific Ocean–Global Atmosphere experiment, decompose the T-IOD into ENSO-forced and internal components [20]. These classifications highlight the diversity of IOD events, but do not focus on their spatial structural variations.

In recent years, a variant of the IOD, termed the IOD Modoki, has been identified based on the SST anomaly pattern of the 1994 event and its comparison with that of 1997 [22]. This variant features a tripole-like structure, with reversed anomalies between the central and western/eastern Indian Ocean. Subsequent studies examined the associated atmospheric and oceanic responses, including changes in the Walker circulation and sea surface height, for both the T-IOD and IOD Modoki events [22,23]. However, the definition of the IOD Modoki in these studies was somewhat arbitrary, relying solely on the 1994 case. Moreover, although a classification method was proposed, no explicit index was provided to quantify the IOD Modoki events.

The Indian Ocean tripole, resembling the IOD Modoki, is derived from the third EOF (EOF3) of tropical Indian Ocean SST anomalies [24]. Zhang *et al*. [24] indicated that the IOT is an independent SST mode of the IOD. The IOT has been linked to climate variations over the western United States, the western Tibetan Plateau, the Australian Monsoon and the warm extremes over the Indochina Peninsula [25–28]. Another SST mode, the southern tropical IOD, is also derived from EOF3 through the simple exclusion of the western Indian Ocean loading; however, the rationale for this exclusion is not explained [29]. Notably, EOF3 accounts for less than 10% of total SST variability, indicating a limited ability to represent observed SST anomaly patterns.

Previous studies have proposed a classification framework for the IOD, comprising the T-IOD and the IOD Modoki. Derived from a single empirical SST mode and one historical case, this framework may not accurately capture the diversity of IOD events and their climate impacts. Here, we identify two types of IOD events based on the centers of SST variability in the tropical Indian Ocean. Both types can be reconstructed using leading EOF modes, indicating that they represent the primary modes of SST variability in the region. We develop corresponding indices, investigate their dynamic characteristics and interdecadal variability, and show that the two IOD types exhibit distinct behaviors. Further, we examine their relationship with East African rainfall and find that one of the defined IOD types exhibits a stronger year-to-year correspondence with East African rainfall than the T-IOD. These results provide a more physically grounded framework for characterizing IOD variability and its regional climatic impacts.

## RESULTS

### Occurrences of the two types of IOD events

**Figure 1.**
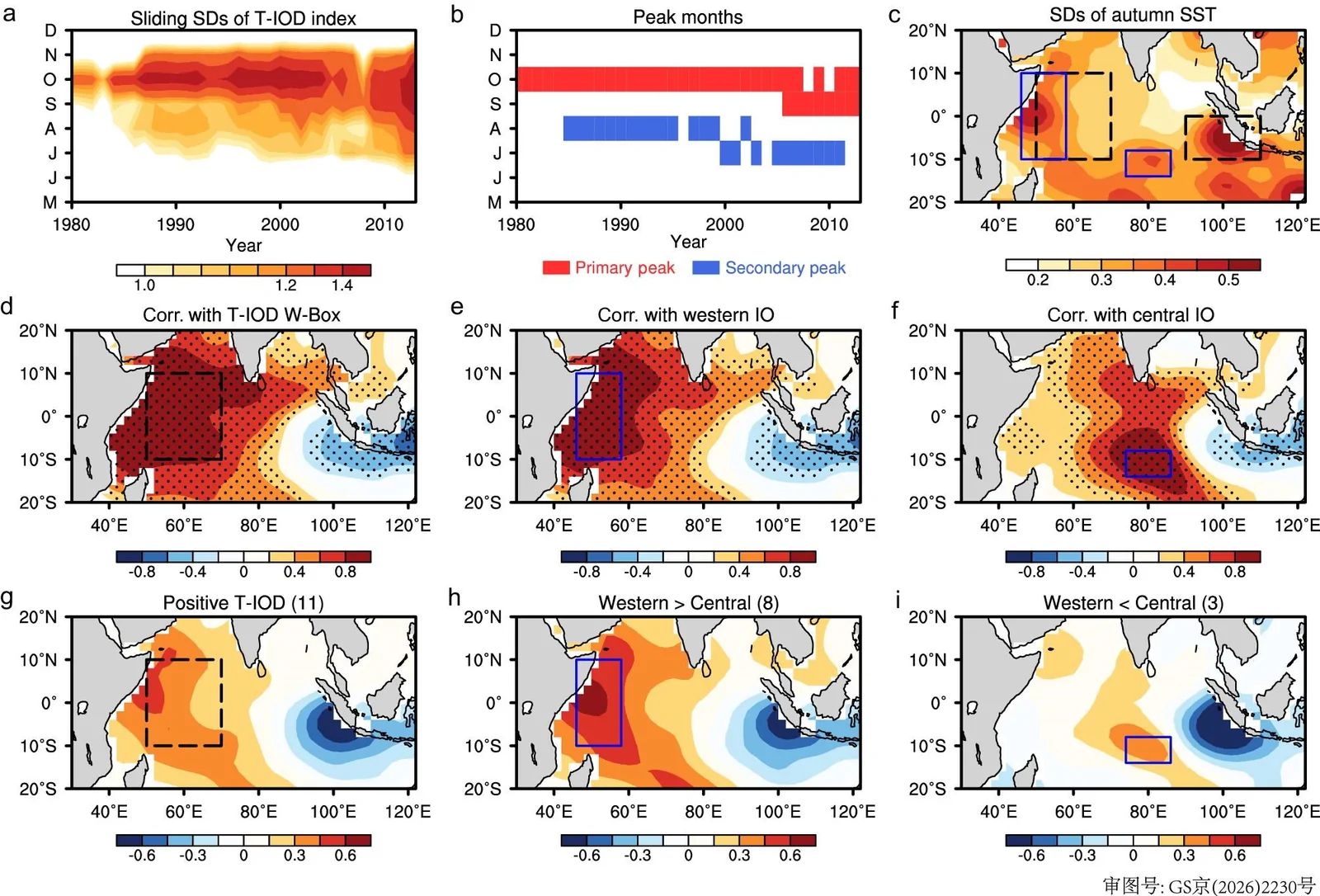
Occurrences of two types of IOD events in boreal autumn. (a–c) Seasonal and spatial limitations of the traditional IOD (T-IOD) index. (a) 21-year sliding standard deviations (shading) of monthly T-IOD index during 1970–2023. (b) Peak months of the standard deviations in each 21-year window, with primary peaks shown in red and secondary peaks shown in blue (see Methods). (c) Standard deviations (°C; shading) of autumn mean SST anomalies during 1970–2023. (d–f) Correlations (shading) between autumn SST and SSTs in the T-IOD western box, the western center, and the central center. Stippling indicates significance at the 95% level. (g–i) Composite SST anomalies (°C; shading) for all positive T-IOD autumns (T-IOD index >0.8) and subsets when the SST anomaly in the western center exceeds or is lower than that in the central center, with event counts indicated in parentheses. In (c–i), black dashed boxes mark the T-IOD index regions, and blue solid boxes indicate the western and/or central Indian Ocean centers (see Methods for exact coordinates). Abbreviations: SDs, standard deviations; W-Box, western box; IO, Indian Ocean.

The T-IOD index, representing the zonal SST anomaly gradient between the western and eastern tropical Indian Ocean, is widely used to study atmospheric and oceanic variability [30–32]. The reliability of such studies depends on how accurately the index captures the spatiotemporal characteristics of SST variability. A key feature of the T-IOD is seasonal-phase locking, with a peak in October [8]. However, 21-year sliding standard deviations reveal a nonstationary seasonal pattern with multiple peaks (Fig. 1a). Although October generally remains the primary peak, in recent decades this peak has extended into September (Fig. 1b). Moreover, several periods exhibit an earlier secondary peak in July or August. These observations indicate that the IOD can peak either in boreal autumn or in summer (July–September), suggesting the diversity of IOD events.

Spatially, the standard deviations of SST anomalies in both boreal autumn and summer reveal three—rather than two—major variability centers across the tropical Indian Ocean: western, central, and eastern (Fig. 1c; Fig. S1a and b). These three SST variability centers are also evident in the OISSTv2 dataset (Fig. S1c and d). The western box used in the T-IOD definition only partially covers the western Indian Ocean variability center (46°E–58°E, 10°S–10°N; hereafter the western center) and also extends toward the central Indian Ocean variability center (74°E–86°E, 14°S–8°S; hereafter the central center), thereby incorporating SST changes from both centers. This spatial mismatch likely limits the T-IOD index’s ability to represent SST variability across the tropical Indian Ocean, as indicated by the elevated residual variance not explained by the T-IOD index in the western and central centers (Fig. S2).

To further explore these variability centers, we examined their interrelationships. SST anomalies in the western and central centers share only ∼8% of their variance during boreal autumn, indicating that they are distinct centers. Correlation maps show that SST anomalies in both the western and central centers are significantly negatively correlated with anomalies in the tropical eastern Indian Ocean, forming two distinct dipole patterns: one between the western and eastern Indian Ocean, and the other between the central and eastern Indian Ocean (Fig. 1e and f). These spatial structures support classifying IOD events into two types: the western–eastern IOD (W-IOD) and the central–eastern IOD (C-IOD).

We then examined SST anomaly patterns using composites of positive T-IOD autumns, defined as cases when the standardized T-IOD index exceeds 0.8. The resulting composite shows the canonical dipole, with warming in the western–central Indian Ocean and cooling in the east (Fig. 1g). However, grouping events by the relative strength of SST anomalies in the western and central centers reveals two patterns: the W-IOD, with warming near the Somali coast (Fig. 1h), and the C-IOD, with warming shifted eastward into the central Indian Ocean (Fig. 1i). Among 11 positive T-IOD autumns, eight are classified as the W-IOD and three as the C-IOD (Fig. S3). Within the eight positive W-IOD events, three also show weaker warming in the central region. The SST distribution during negative T-IOD autumns can also be categorized into two distinct types (Fig. S4). These suggest that a substantial portion of T-IOD events exhibit characteristics of the C-IOD pattern.

A parallel analysis for boreal summer, when the T-IOD index shows an earlier secondary peak, also reveals two distinct dipoles in correlation maps and composites (Fig. S5). SST anomalies in the western and central centers are significantly negatively correlated with those in the eastern tropical Indian Ocean, and summer composites also confirm two spatially distinct patterns. About half (7 of 15) of positive T-IOD summers exhibit the C-IOD pattern (Fig. S6), including the 2019 super IOD summer. These results support the existence of two IOD types in both boreal autumn and summer, which may differ in variability and climatic impacts historically and under future scenarios.

### Identification of the two types of IOD

**Figure 2.**
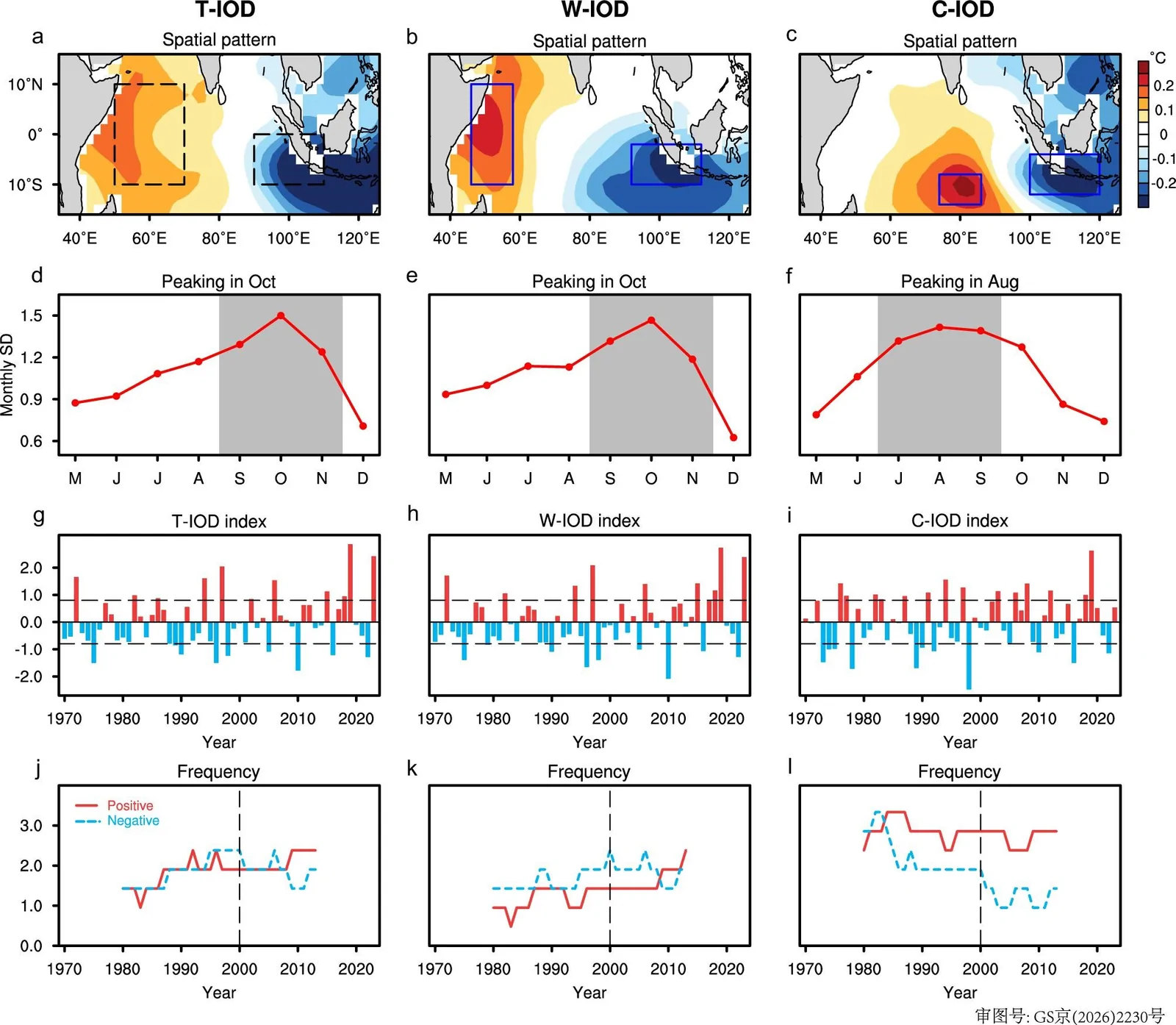
Definition and spatiotemporal characteristics of two IOD types. (a–c) Spatial patterns of SST anomalies (°C, shading) for the W-IOD and C-IOD, in comparison with the T-IOD. The T-IOD corresponds to EOF2, while the W-IOD and C-IOD are reconstructed as the linear combinations (EOF2 + EOF3)/$\sqrt {\mathrm{2}} $ and (EOF2 − EOF3)/$\sqrt {\mathrm{2}} $, respectively. Rectangles indicate the regions used to calculate the indices (see Methods for exact coordinates). (d–f) Monthly standard deviations of the T-IOD, W-IOD, and C-IOD indices from 1970 to 2023. (g–i) Time series of standardized autumn mean T-IOD, autumn mean W-IOD, and summer mean C-IOD indices from 1970 to 2023, with dashed reference lines at ±0.8. (j–l) 21-year running mean occurrence frequency (events per decade) of T-IOD, W-IOD, and C-IOD events, defined as index values exceeding ±0.8 during their respective peak seasons. Positive- and negative-phase frequencies are shown by red solid and blue dashed lines, respectively. Abbreviation: EOF, empirical orthogonal function.

Objectively defining the two types of IOD events and quantifying their intensity and climatic impacts remains challenging. To address this, we extracted the leading modes of SST anomalies over the tropical Indian Ocean (34°E–126°E, 16°S–16°N) using EOF analysis (Fig. S7). EOF1 reveals basin-wide warming, commonly referred to as the Indian Ocean Basin Mode [33]. EOF2 exhibits a west–east dipole corresponding to the T-IOD (Fig. 2a) [8], while EOF3 displays a tripole with strong SST contrast between the western and central Indian Ocean, with its western Indian Ocean loading overlapping with that of EOF2. The variability associated with EOF2 and EOF3 also exhibits a lead–lag relationship (Fig. S8), suggesting a temporal linkage between the two modes. By combining EOF2 and EOF3 (see Methods), (EOF2 + EOF3)/$\sqrt {\mathrm{2}} $ reproduces the W-IOD pattern, with pronounced warming in the west and cooling in the east (Fig. 2b), whereas (EOF2 − EOF3)/$\sqrt {\mathrm{2}} $ yields the C-IOD pattern, with positive anomalies in the central Indian Ocean and negative anomalies in the east, but no signal in the west (Fig. 2c). These two IOD patterns are supported by the maximum negative response analysis method, a point-to-point approach developed in this study that identifies the strongest SST responses between two regions (see Methods and Fig. S9). They are also reproduced using the EOF combination method based on the OISSTv2 dataset (Fig. S10). These results further support the robustness of the identified IOD patterns.

Accordingly, the two types of IOD indices can be calculated using two approaches. The first derives the indices from combinations of the principal components (PC2 and PC3) associated with EOF2 and EOF3 (see Methods), defining the W-IOD and C-IOD indices as (PC2 + PC3)/$\sqrt {\mathrm{2}} $ and (PC2 − PC3)/$\sqrt {\mathrm{2}} $, respectively. The second follows the method used for the T-IOD index [8], defining the W-IOD index as the SST anomaly difference between the western center and eastern Indian Ocean region 1 (92°E–112°E, 10°S–2°S; Fig. 2b) and the C-IOD index as the difference between the central center and eastern Indian Ocean region 2 (100°E–120°E, 12°S–4°S; Fig. 2c). Both approaches produce highly consistent results (Fig. S11). For simplicity and broader applicability, indices based on SST anomaly differences are used in the following analysis.

In addition to their distinct spatial patterns, the W-IOD and the C-IOD differ in seasonality and variability. The W-IOD peaks in boreal autumn, consistent with the T-IOD, whereas the C-IOD peaks earlier, during boreal summer (July–September) (Fig. 2d–f), helping to explain the nonstationary phase locking of the T-IOD index. They also differ in interannual variability and event frequency (Fig. 2g–i). By defining IOD events as those with absolute standardized indices exceeding 0.8 during their respective peak seasons, we identify 18 W-IOD, 20 T-IOD, and 25 C-IOD events (see Table S1 for the event years). Of the 18 W-IOD events, 17 coincide with the T-IOD events, showing strong temporal overlap. By contrast, C-IOD and W-IOD exhibit distinct interannual variability. Of the 25 C-IOD events, 13 occur in years without a W-IOD (JAS for C-IOD, SON for W-IOD), while 6 of the 18 W-IOD events occur without a C-IOD. Twelve years feature both C-IOD and W-IOD events. Although they occasionally co-occur in the same year, each type also occurs on its own in many cases (Fig. S12), reinforcing their classification as two distinct modes.

We next examine interdecadal changes in the frequency of the two IOD types, separated into positive and negative phases. Compared with the moderate increase in positive T-IOD events (Fig. 2j), the 21-year running frequency of positive W-IOD events shows a clearer upward shift, rising by ∼0.5 events per decade around 2000 (Fig. 2k and Table S2). By contrast, the frequency of negative C-IOD events declines sharply, with an interdecadal decrease of ∼1.0 events per decade over the same period (Fig. 2l and Table S2). Frequencies of negative W-IOD and positive C-IOD events remain relatively stable. Taken together, these differences in spatial patterns, seasonality, and frequency indicate that the two IOD types are likely governed by distinct physical mechanisms.

### Distinct ocean–atmosphere coupling in the two types of IOD events

**Figure 3.**
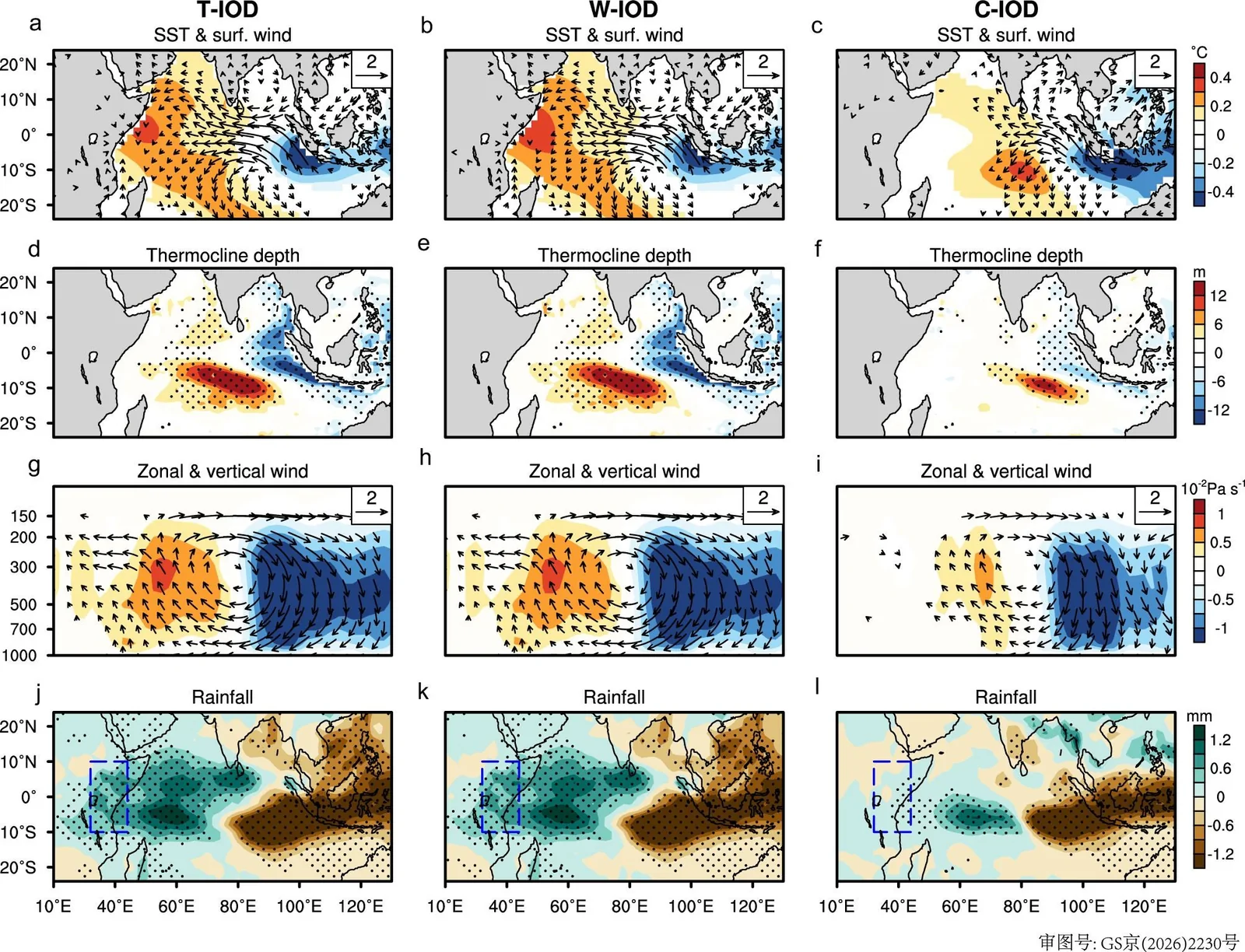
Contrasting Bjerknes feedback associated with two types of IOD. Regression patterns onto the W-IOD (middle) and C-IOD (right) indices during 1970–2023, with those onto the T-IOD index (left) shown for comparison. (a–c) SST anomalies (°C, shading) with near-surface (σ = 0.995) wind anomalies (m s^−1^, vectors). (d–f) Thermocline depth anomalies (m, shading). (g–i) Vertical velocity anomalies (10^−2^ Pa s^−1^, shading; positive upward), overlaid with zonal and vertical wind anomalies (m s^−1^ and 10^−2^ Pa s^−1^, vectors), meridionally averaged over 12.5°S–0°. (j–l) Rainfall anomalies (mm, shading); the rectangles indicate the East African region (32°E–44°E, 10°S–10°N). Shown anomalies in (a–c) and (g–i) and stippling in (d–f) and (j–l) indicate significance at the 95% confidence level.

Both IOD types are governed by ocean–atmosphere interactions via the Bjerknes feedback, as previously established for the T-IOD [8]. The W-IOD shares similar feedbacks with the T-IOD, whereas the C-IOD differs in spatial patterns and temporal evolution (Fig. 3 and Fig. S13). At the peak season of positive W-IOD events, the equatorial Indian Ocean exhibits a pronounced zonal gradient of SST anomalies between the western Indian Ocean off Somalia and the eastern Indian Ocean, accompanied by anomalous easterlies along the equator (Fig. 3b). These easterlies drive warm surface waters westward, causing their accumulation in the western Indian Ocean. This process explains why the western Indian Ocean, rather than the broader western–central basin, serves as the key center of SST variability.

As the easterlies reach the central–western Indian Ocean, they bifurcate northward and southward, forming large-scale anticyclonic-like circulations. The resulting wind stress curl drives Ekman downwelling, deepening the thermocline in the central–western basin, especially its southern sector. Meanwhile, westward surface currents intensify upwelling in the eastern Indian Ocean, shoaling the thermocline (Fig. 3e). These opposing thermocline anomalies amplify the zonal SST anomaly gradient through oceanic processes: a deepened thermocline in the central–west suppresses cold-water entrainment and enhances westward surface heat transport, whereas a shoaled thermocline in the east enhances upwelling and surface cooling.

In the atmosphere, the SST anomaly pattern of positive W-IOD events induces an anomalous Walker circulation, with ascent over the western Indian Ocean and descent over the east (Fig. 3h). This circulation enhances convection in the west and suppresses it in the east (Fig. 3k). The associated diabatic heating anomalies generate anticyclonic-like responses and equatorial easterly anomalies via a Gill-type response, which in turn reinforce the surface wind and oceanic anomalies. These coupled ocean–atmosphere feedbacks establish a positive feedback loop that sustains and amplifies the W-IOD, analogous to the T-IOD (Fig. 3a, d, g and j).

During the peak season of C-IOD events, warm SST anomalies are concentrated in the central Indian Ocean, accompanied by anomalous easterlies over the central–eastern sector (Fig. 3c). The associated anticyclonic circulation induces only modest thermocline deepening in the southern central–eastern basin (Fig. 3f). The resulting zonal SST anomaly gradient drives a weaker Walker circulation, with ascent over the central basin and descent in the east, enhancing convection over the central Indian Ocean rather than over the western basin (Fig. 3i and l). These relatively weak ocean–atmosphere anomalies result in a subdued Bjerknes feedback during the C-IOD events.

A key question is what controls the differences in Bjerknes feedback between the two types of IOD. In both types, cool SST anomalies and anomalous easterlies first emerge in the eastern Indian Ocean (Fig. S13). For the W-IOD, warm SST anomalies subsequently develop in the west by July–August, accompanied by easterly anomalies that extend across the Indian Ocean, promoting the accumulation of warm water in the west and the westward extension of the enhanced Rossby wave signals (Fig. S14). During this stage, the easterly anomalies also weaken the climatological southwest monsoon winds, leading to a reduction in the Somali upwelling, which further enhances SST in the western Indian Ocean [34]. By contrast, during the C-IOD, easterly anomalies shift to northerlies over the central Indian Ocean by May–June, establishing an anticyclonic circulation that favors warming there. These contrasts in the timing and spatial evolution of wind anomalies may play a key role in modulating both the onset and the location of positive SST anomalies in the two types of IOD. These processes require further validation and analysis in future studies.

### Diverse impacts of the two types of IOD on east African rainfall

Previous studies have shown that the T-IOD significantly affects East African rainfall, strongly enhancing it during positive phases and reducing it during negative phases [8]. Considering the presence of two distinct IOD types, we further examine their respective impacts during their peak seasons and reveal marked contrasts. The W-IOD generates strong rainfall anomalies over East Africa, closely resembling those associated with the T-IOD. In contrast, the C-IOD has a negligible effect, with anomalies near zero. The distinct relationships between the two IOD patterns and East African rainfall are also verified by Singular Value Decomposition (SVD) analysis (Fig. S15). These differences primarily arise from the contrasting convective patterns of the two IOD types.

Both the W-IOD and the T-IOD strongly influence autumn rainfall in East Africa (32°E–44°E, 10°S–10°N), with comparable correlation coefficients of 0.83 and 0.82. However, their year-to-year correspondence with East African rainfall could differ markedly, as T-IOD events can feature SST anomalies in either the western or central Indian Ocean (Fig. 1h and i). To examine this, W-IOD and T-IOD events are defined when the standardized autumn index exceeds ±0.8, while East African rainfall events are identified when autumn mean rainfall exceeds ±0.5, acknowledging that East African rainfall variability is also influenced by factors beyond the IOD.

**Figure 4.**
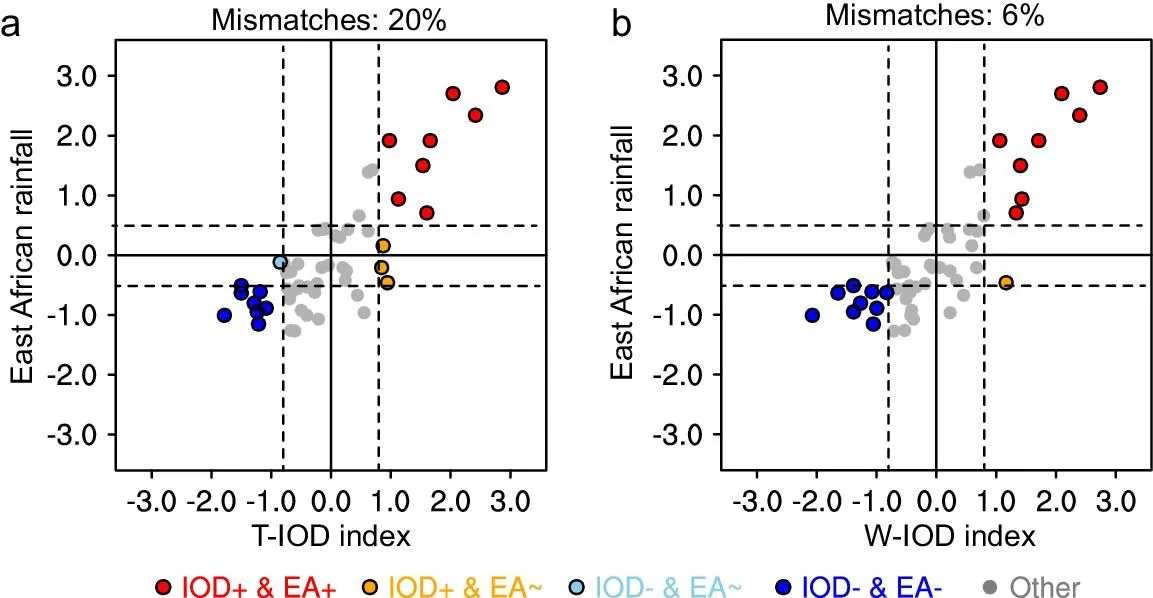
The W-IOD shows a stronger correspondence with East African rainfall than the T-IOD. Scatter plots show the standardized autumn T-IOD index (a) and W-IOD index (b) against autumn East African rainfall (32°E–44°E, 10°S–10°N). IOD events are classified as positive (IOD+) or negative (IOD–) when the index exceeds 0.8 or falls below −0.8, respectively. East African rainfall events are categorized as positive (EA+, >0.5), negative (EA–, <−0.5), or near-normal (EA∼, −0.5 to 0.5) based on standardized anomalies. Dot colors indicate combinations of IOD polarity and rainfall response (see legend). In (a), mismatched years (when rainfall does not follow the expected change under IOD events) are 1986, 2002, and 2018 (orange dots) and 1989 (light blue dot). In (b), the only mismatched year is 2018 (orange dot); additionally, the W-IOD identifies another matched year, 1979 (extra blue dot). Overall, ∼20% of T-IOD events (4 out of 20) show poor correspondence with East African rainfall, compared to just one (∼6%) for the W-IOD.

Among the 20 identified T-IOD events, 16 are matched with the expected East African rainfall anomalies: eight positive with increased rainfall and eight negative with decreased rainfall (Fig. 4a). The remaining four show near-normal rainfall: two are pure C-IOD events, one exhibits a complex SST anomaly pattern, and the fourth is the unusual 2018 case (Fig. S16). In the 2018 event, the expected anomalous Walker circulation linked to the warmer SST in the western Indian Ocean was likely disrupted by strong cold conditions to the northwest of Australia. Overall, 80% of T-IOD events coincide with significant East African rainfall anomalies, while 20% do not, implying the risk of false alarms if forecasts rely solely on the T-IOD signal.

In comparison, the W-IOD exhibits stronger correspondence, with 17 matched years and only one mismatch (Fig. 4b). Sixteen of these coincide with the T-IOD, while the W-IOD additionally captures the 1979 negative event accompanied by suppressed East African rainfall (Fig. S17). By excluding the three mismatches under the T-IOD, the W-IOD reduces the false alarm rate from 20% to ∼6%, the only exception being the unusual 2018 event. Overall, the W-IOD provides a more consistent representation of East African rainfall variability, reinforcing the importance of classifying IOD events into two types.

Besides, the interdecadal variability of the IOD further informs the interdecadal shift of East African rainfall. The 21-year running mean frequency of positive T-IOD events has increased moderately (Fig. 2j), suggesting a modest rise in IOD-enhanced rainfall over East Africa. However, this T-IOD–based assessment may underestimate the actual increase. Positive W-IOD events, which correspond more closely with autumn rainfall over East Africa, have increased more sharply (Fig. 2k). This pronounced increase poses an escalating threat to East African countries vulnerable to heavy rainfall and underscores the urgent need to strengthen disaster preparedness and management.

## DISCUSSION

One may ask why the IOD is divided into W-IOD and C-IOD, rather than into T-IOD and IOD Modoki as proposed by Endo and Tozuka [22]. First, the T-IOD fails to capture the major SST anomaly centers, thereby mixing distinct event types. Second, SST anomalies in the central and western regions are not significantly negatively correlated (Fig. 1e and f), questioning the tripole-like structure of the IOD Modoki. Third, the Bjerknes feedback of the IOD Modoki essentially mirrors that of the C-IOD but at weaker strength (Fig. S18), suggesting that the C-IOD provides a more physically grounded representation. Finally, the IOD Modoki, originally identified from the 1994 SST anomaly, cannot account for the enhanced East African rainfall that year, since it exerts little influence on the rainfall in East Africa [22]. Conversely, this rainfall is naturally accounted for under the W-IOD and C-IOD framework, particularly the positive W-IOD phase (Fig. S19).

Consistent with interdecadal shifts in the Pacific and Atlantic since the 2000s, the W-IOD and C-IOD exhibit pronounced interdecadal variability, which is not captured by the T-IOD. Positive W-IOD events have increased significantly, whereas negative C-IOD events have declined markedly (Fig. 3k and l). A potential driver of this shift is a deepening of the central Indian Ocean thermocline since 2000 (Fig. S20), which may have suppressed negative C-IOD events while favoring positive W-IOD events. The causes of this thermocline change remain uncertain but could involve variations in ocean heat content, wind forcing (locally or remotely induced by SST anomalies), and the Indonesian Throughflow. Further work is needed to elucidate these mechanisms and their influence on IOD variability.

**Figure 5.**
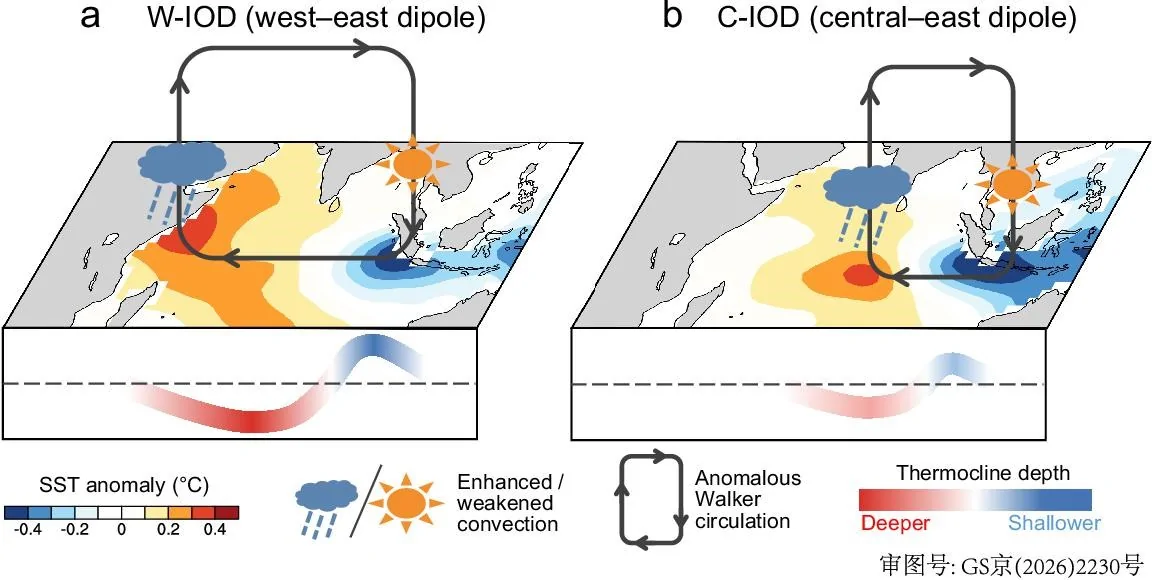
Conceptual model illustrating the distinct characteristics, physical processes, and climate impacts of the W-IOD (a) and C-IOD (b).

Overall, our results underscore the importance of distinguishing two distinct types of IOD, which differ in spatial patterns, seasonal evolution, ocean–atmosphere processes, and impacts on East African rainfall (Fig. 5). To facilitate comparison, a comprehensive summary of these two IOD types and existing classifications is provided in Table S1. Compared with traditional classifications, this approach offers a more precise representation of Indian Ocean SST variability and its climate impacts. Importantly, the classification of the IOD presented here is grounded in clear physical reasoning, rather than arbitrary criteria. Furthermore, the two IOD types may exert distinct remote influences, exhibit different interactions with ENSO (Fig. S21) and extratropical processes, and potentially respond differently to future climate change. This new classification framework provides a pathway to revisit past findings and guide future research, thereby enhancing understanding of Indian Ocean variability and improving climate prediction, projections, and risk management in affected regions.

## METHODS

### Observational data and preprocessing

Monthly SST data were obtained from the National Oceanic and Atmospheric Administration (NOAA) Extended Reconstructed SST version 5 (ERSSTv5) with a spatial resolution of 2° × 2° [35]. The NOAA Optimum Interpolation Sea Surface Temperature version 2 (OISSTv2) dataset with a spatial resolution of 0.25° × 0.25°, available from September 1981 [36], was used for additional validation over the period 1982–2023. Monthly atmospheric variables were obtained from the National Centers for Environmental Prediction (NCEP) Reanalysis I at 2.5° × 2.5° resolution [37]. Monthly thermocline depth data (the 20 °C isotherm) were obtained from the European Centre for Medium-Range Weather Forecasts (ECMWF) Ocean Reanalysis System 5 (ORAS5) at ∼0.25° × 0.25° resolution [38]. Monthly rainfall data were obtained from the fifth generation ECMWF atmospheric reanalysis (ERA5) at 0.25° × 0.25° resolution [39].

Among these datasets, the OISSTv2 SST and monthly thermocline depth data were bilinearly interpolated to 1° × 1° resolution, whereas rainfall data were conservatively remapped to 2° × 2° resolution. The analysis period spans 1970–2023, providing a sufficiently long record to examine interdecadal shifts. All datasets had their annual cycle removed and were linearly detrended prior to analysis.

### Analysis methods

To identify key centers of SST variability, the standard deviation of SST anomalies was calculated. Composite analysis was conducted to reveal SST anomaly patterns corresponding to different IOD event categories. The EOF combination method was used to define two types of IOD, and a maximum negative response analysis method was developed to verify these definitions (see below for details). Regression and correlation analyses were performed to examine atmospheric and oceanic variables associated with the indices, and SVD analysis was also applied to examine coupled variability between SST and rainfall fields. The statistical significance of regression and correlation coefficients was assessed using a two-tailed Student’s *t*-test.

### Regional definitions

The T-IOD index is defined as the difference in SST anomalies between the western box and eastern box of the equatorial Indian Ocean:

- Western box: 50°E–70°E, 10°S–10°N;
- Eastern box: 90°E–110°E, 10°S–0°.

For analyses of the major centers of Indian Ocean SST variability and for calculating the W-IOD and C-IOD indices using SST anomaly differences:

- Western center: 46°E–58°E, 10°S–10°N;
- Central center: 74°E–86°E, 14°S–8°S;
- Eastern Indian Ocean region 1: 92°E–112°E, 10°S–2°S;
- Eastern Indian Ocean region 2: 100°E–120°E, 12°S–4°S.

For East African rainfall analyses:

- East Africa region: 32°E–44°E, 10°S–10°N.

### Seasonal definitions

Boreal summer and autumn in this study are defined as July–September and September–November, respectively. Seasonal means of SST and rainfall are calculated accordingly.

### Peak identification

To determine the timing of peak variability, we calculated the monthly standard deviation of the T-IOD index using a 21-year sliding window. A month was defined as a peak if its standard deviation increased relative to the previous month and was followed by either a decrease or a marginal change (±5%) in the subsequent month. This criterion captures both sharp and plateau-like peaks, highlighting deviations from the pronounced monotonic increase in the T-IOD variability prior to October (Fig. S22). Within each window, the primary peak comprises the month with the highest standard deviation and any peaks in adjacent months, whereas all other peaks are regarded as secondary.

### EOF combination method and associated index calculation

To reconstruct SST anomaly patterns for the two IOD types, the EOF method was applied to monthly SST anomalies in the tropical Indian Ocean. The resulting PCs were normalized by their respective standard deviations and used to scale the corresponding EOFs. The combined pattern (EOF2 + EOF3)/$\sqrt {\mathrm{2}} $ represents the W-IOD, whereas (EOF2–EOF3)/$\sqrt {\mathrm{2}} $ corresponds to the C-IOD. Accordingly, the W-IOD and C-IOD indices are defined as (PC2 + PC3)/$\sqrt {\mathrm{2}} $ and (PC2–PC3)/$\sqrt {\mathrm{2}} $, respectively. These definitions ensure that the sum of the products of the two IOD patterns equals that of the two EOF modes, i.e. (EOF2 + EOF3)/$\sqrt {\mathrm{2}} $ × (PC2 + PC3)/$\sqrt {\mathrm{2}} $ + (EOF2–EOF3)/$\sqrt {\mathrm{2}} $ × (PC2–PC3)/$\sqrt {\mathrm{2}} $ = EOF2 × PC2 + EOF3 × PC3. A similar EOF-based approach has been used to reconstruct two types of ENSO and the Atlantic Niño, successfully capturing the key SST patterns in both the Atlantic and Pacific Oceans [7,14].

### Maximum negative response analysis method

The maximum negative response analysis is a point-to-point approach developed in this study to quantify the largest negative response between SST anomalies in two regions. This approach is inspired by the teleconnection method proposed by Wallace and Gutzler [40], with the correlation coefficient scaled by the local standard deviation to quantify the magnitude of the negative response. The method involves the following steps. One region is defined as the reference region, and the other as the target region. For each grid point in the target region, correlations with all grid points in the reference region are calculated. The most negative correlation is then identified and multiplied by the standard deviation of the target point to quantify the maximum negative response. Repeating this procedure for all points in the target region produces a spatial map of maximum negative responses. When two regions exhibit mutually strong negative responses, they are considered to form a dipole structure.

## Supplementary Material

nwag519_Supplemental_File

## Data Availability

All datasets used in this study are publicly accessible: monthly SST from the NOAA ERSSTv5, https://psl.noaa.gov/data/gridded/data.noaa.ersst.v5.html; monthly SST from the NOAA OISSTv2, https://psl.noaa.gov/data/gridded/data.noaa.oisst.v2.highres.html; monthly atmospheric variables from the NCEP Reanalysis I, https://psl.noaa.gov/data/gridded/data.ncep.reanalysis.html; monthly thermocline depth from the ORAS5, https://cds.climate.copernicus.eu/datasets/reanalysis-oras5?tab=download; and monthly rainfall from the ECMWF ERA5, https://cds.climate.copernicus.eu/datasets/reanalysis-era5-single-levels-monthly-means?tab=download. The custom codes used for data analysis are available from the corresponding author upon reasonable request.
