# Supplementary material for: Two distinct types of the Indian Ocean Dipole
Source: Natl Sci Rev. 2026 Aug 21;13(18):nwag519. doi: 10.1093/nsr/nwag519 (PMC13615526; doi:10.1093/nsr/nwag519)
Supplement: Supplementary file 1 [file nwag519_supplemental_file.pdf]

Supporting Information for

Two distinct types of the Indian Ocean Dipole

Jiayu Zheng[1,2,3] and Chunzai Wang[1,2,3,*]

[1]Laboratory of Tropical Oceanography, South China Sea Institute of Oceanology, Chinese Academy of Sciences, Guangzhou, China

[2]Global Ocean and Climate Research Center, South China Sea Institute of Oceanology, Chinese Academy of Sciences, Guangzhou, China

[3]China-Sri Lanka Belt and Road Joint Laboratory on Tropical Oceanography, South China Sea Institute of Oceanology, Chinese Academy of Sciences, Guangzhou, China

*Corresponding author. E-mail: cwang@scsio.ac.cn

This PDF file includes:

Figures S1–S22

Tables S1–S2

Figures

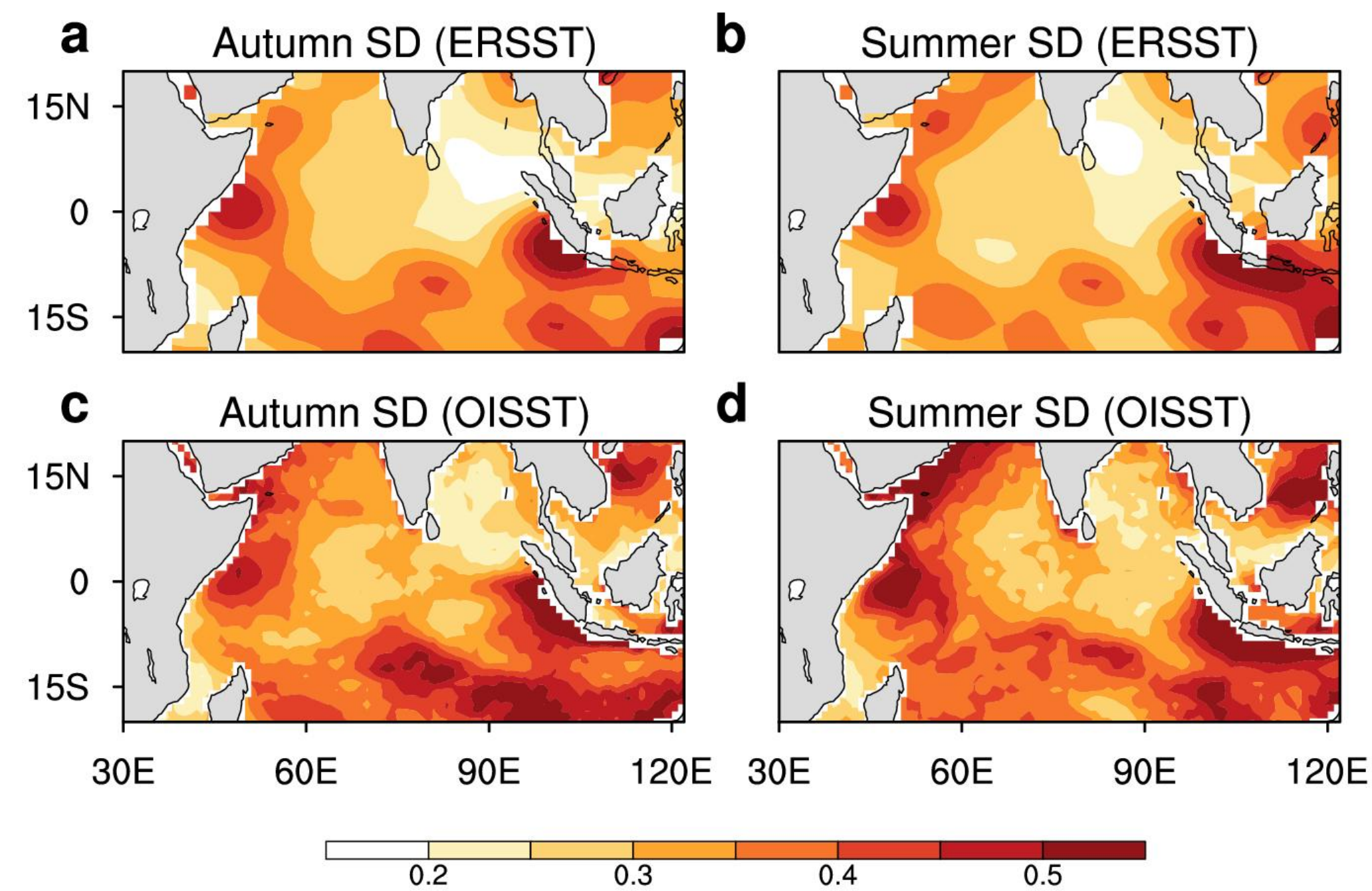

Figure S1. The SST variability during boreal autumn and summer exhibits three major variability centers. Standard deviations (°C, shading) of seasonal mean SST anomalies during 1970–2023 based on (a, b) ERSSTv5 and (c, d) OISSTv2. Boreal autumn and summer are defined as September–November and July–September, respectively. Abbreviation: SDs, standard deviations.

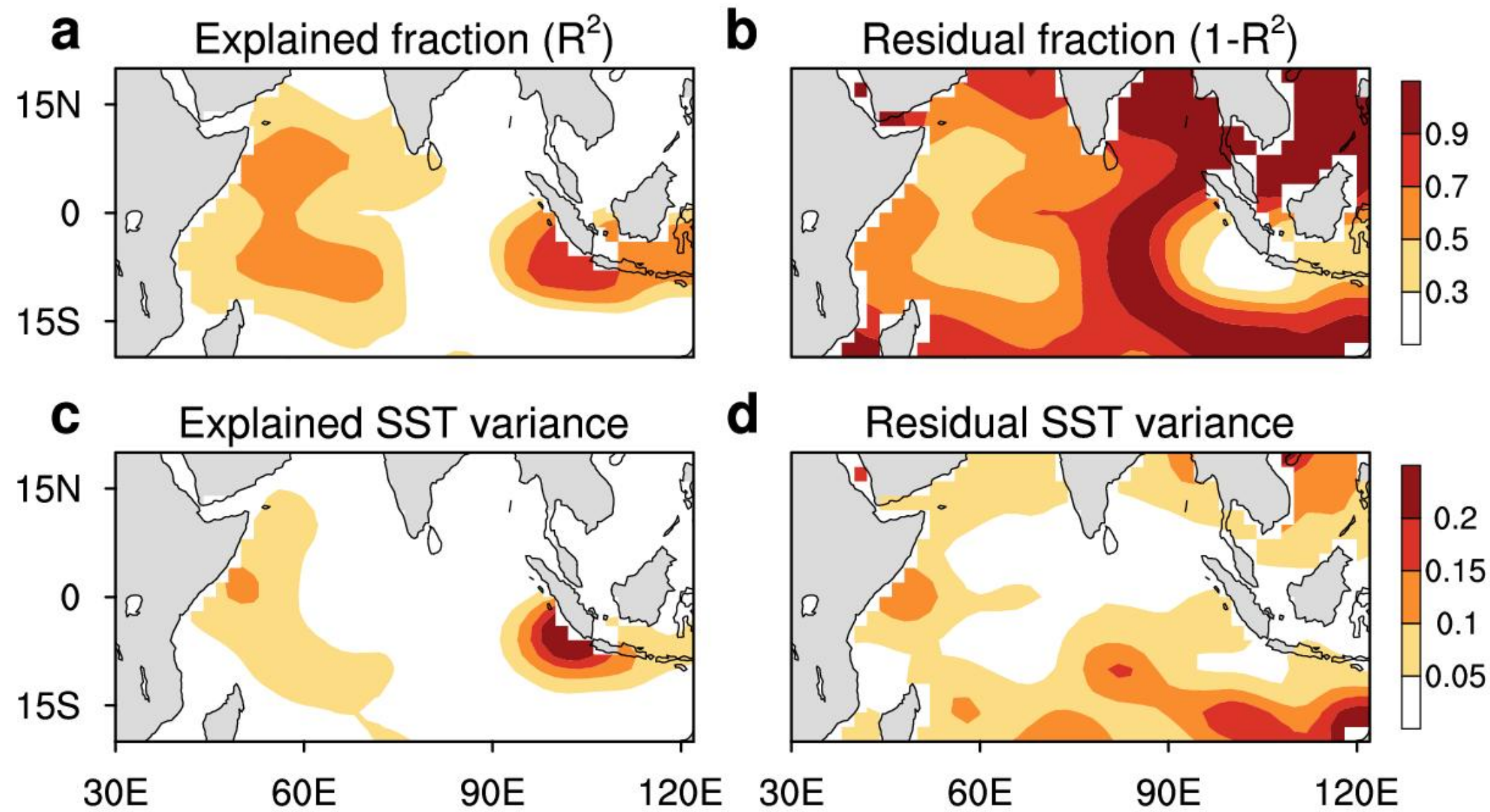

Figure S2. Explained and residual SST variance associated with the T-IOD index. (a) Explained variance fraction (R^2); (b) residual variance fraction ($1-R^2$); (c) explained SST variance (units: °C^2); and (d) residual SST variance (units: °C^2). The explained and residual SST variances are calculated by multiplying the total SST variance by R^2 and $1-R^2$, respectively.

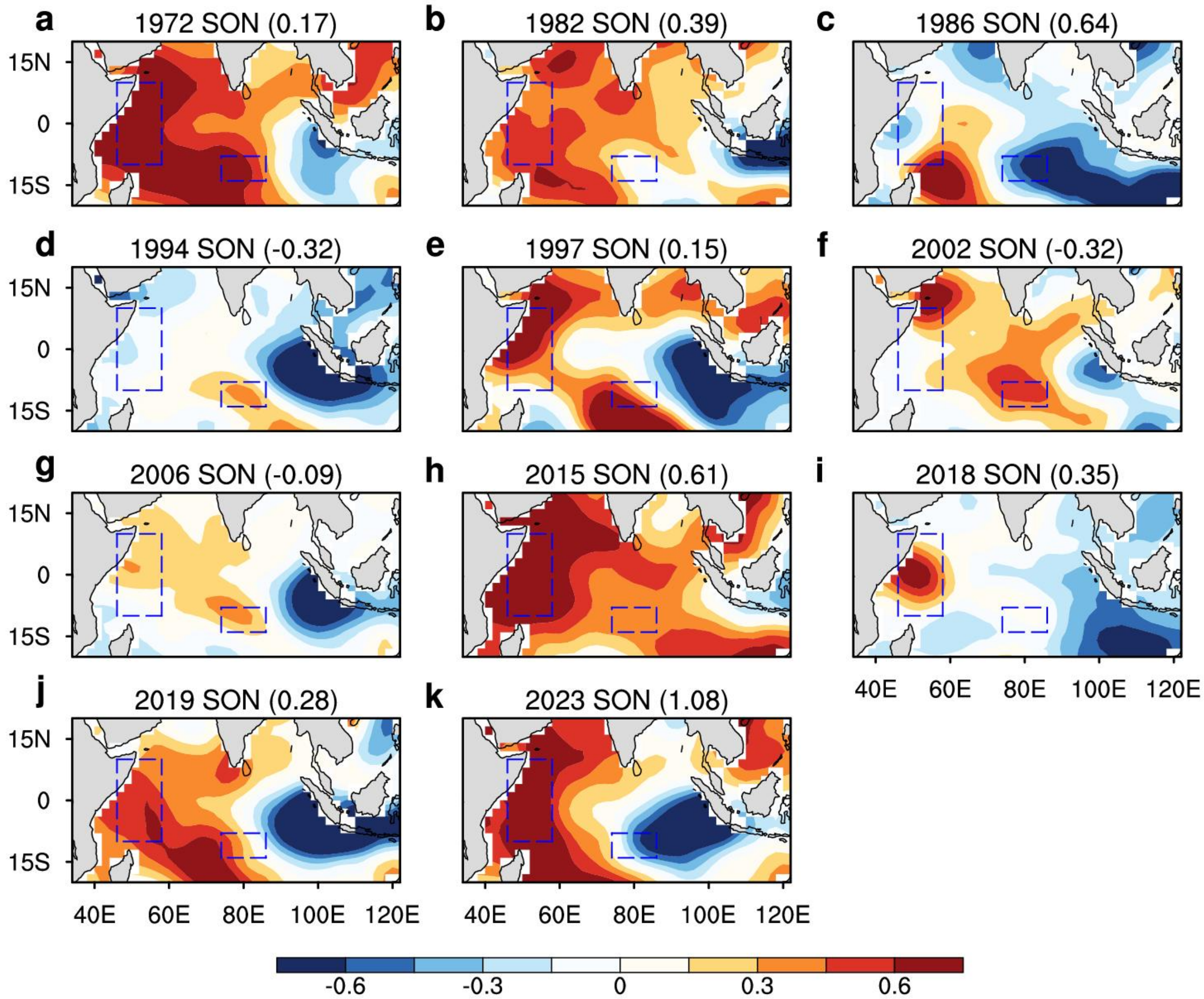

Figure S3. SST anomaly patterns during positive T-IOD autumns. SST anomalies (°C, shading) for positive T-IOD autumns, defined by a standardized autumn mean T-IOD index > 0.8. Blue dashed boxes mark the western and central variability centers (see Methods for exact coordinates). Values in parentheses denote the SST anomaly difference between these centers. Based on this difference, eight autumns are classified as the W-IOD and three as the C-IOD. Abbreviation: SON, September–November.

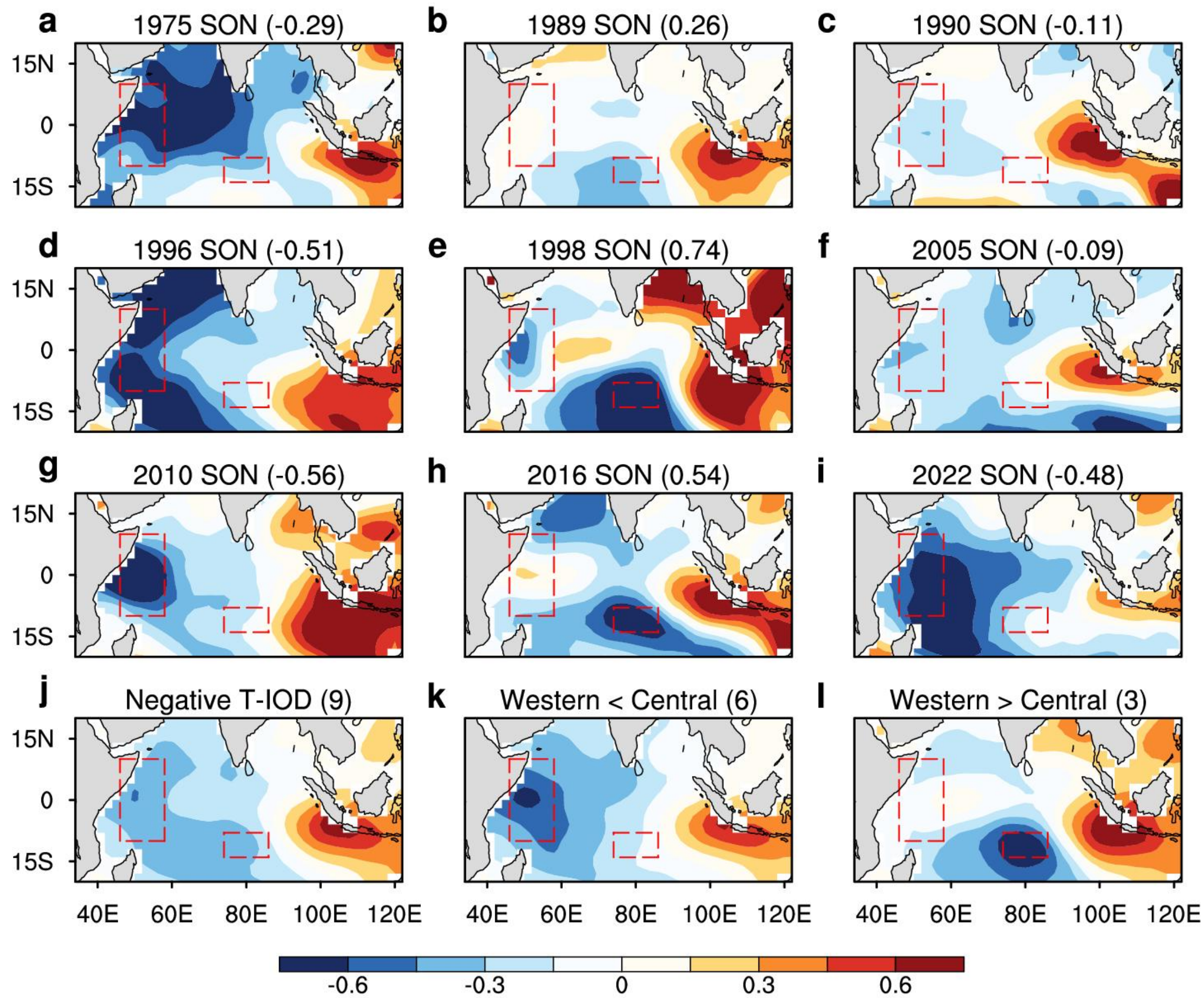

Figure S4. SST anomaly patterns during negative T-IOD autumns. (a–i) SST anomalies (°C, shading) for positive T-IOD autumns, defined by a standardized autumn mean T-IOD index < -0.8. (j–l) Composite SST anomalies (°C, shading) for all positive T-IOD autumns and subsets where the SST anomaly in the western center is lower than or exceeds that in the central center. Red dashed boxes mark the western and central variability centers (see Methods for exact coordinates). Values in parentheses in (a–i) denote the SST anomaly difference between the two centers, while values in parentheses in (j–l) indicate event counts. Based on the SST anomaly differences, six autumns are classified as the W-IOD and three as the C-IOD. Abbreviation: SON, September–November.

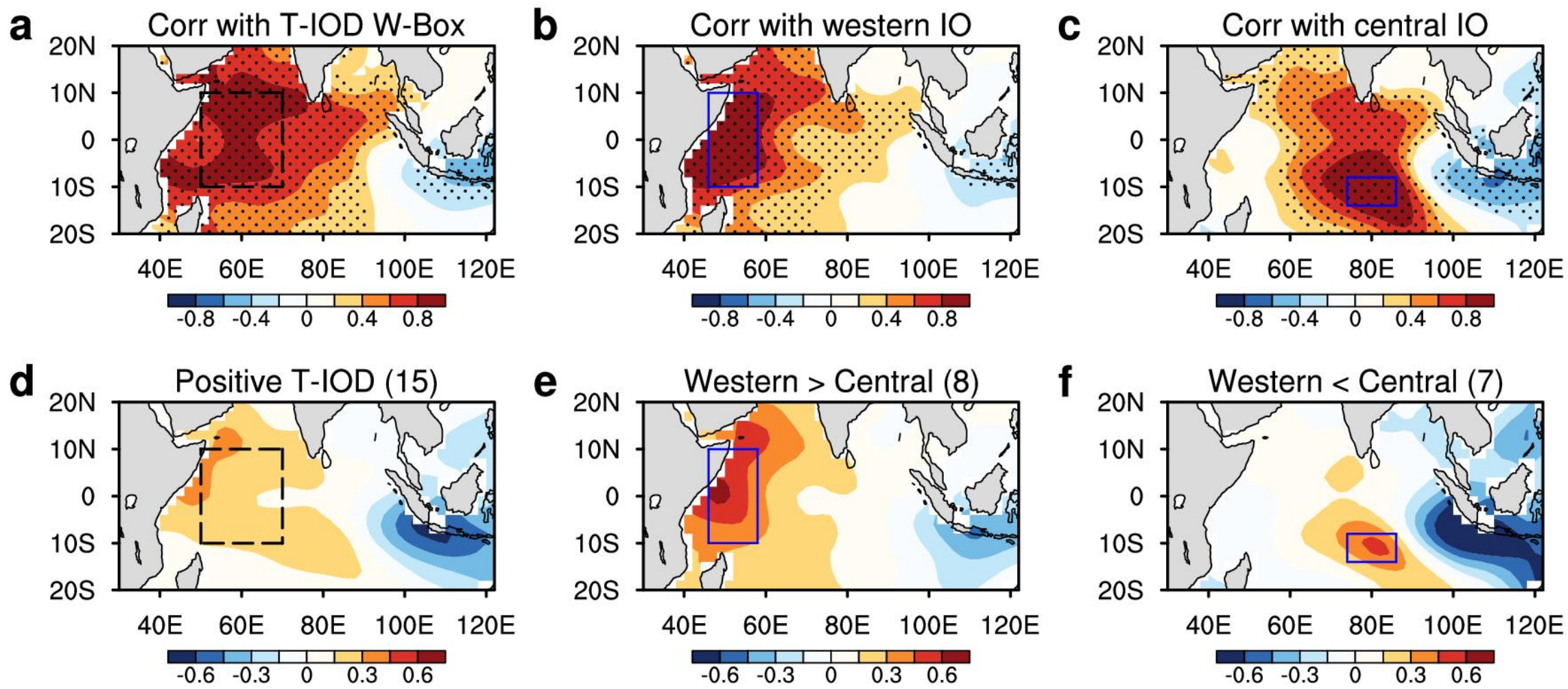

Figure S5. Occurrences of two types of IOD events in boreal summer. (a–f) As in Fig. 1d–i, but for July–September. Correlation maps and composites indicate the presence of two IOD types in boreal summer. In addition, C-IOD events account for a larger proportion of T-IOD events in boreal summer than in autumn.

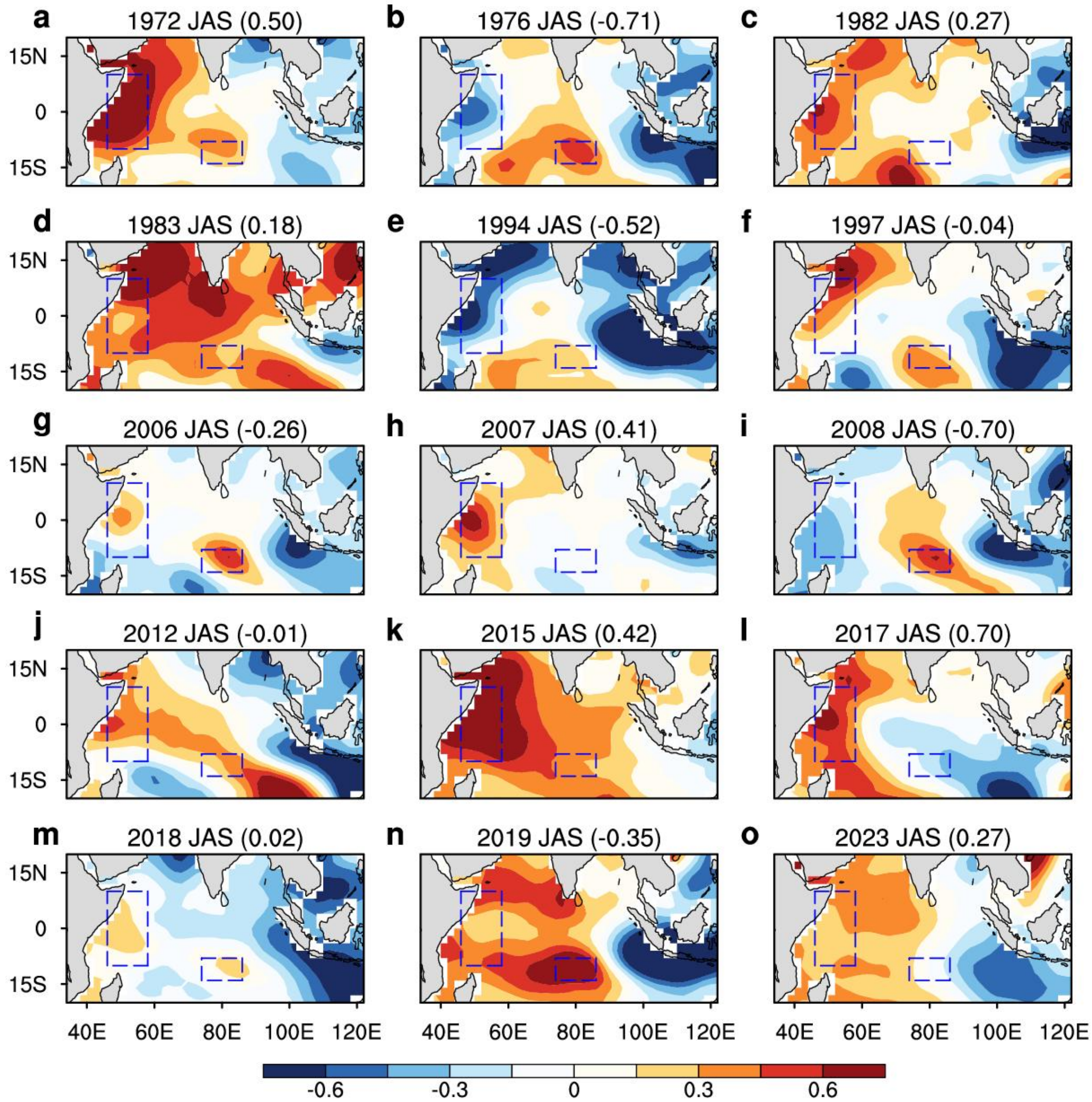

Figure S6. As in Figure S3, but for positive T-IOD summers. Based on the SST anomaly difference between the western and central centers, eight summers are classified as the W-IOD and seven as the C-IOD. Abbreviation: JAS (July–September).

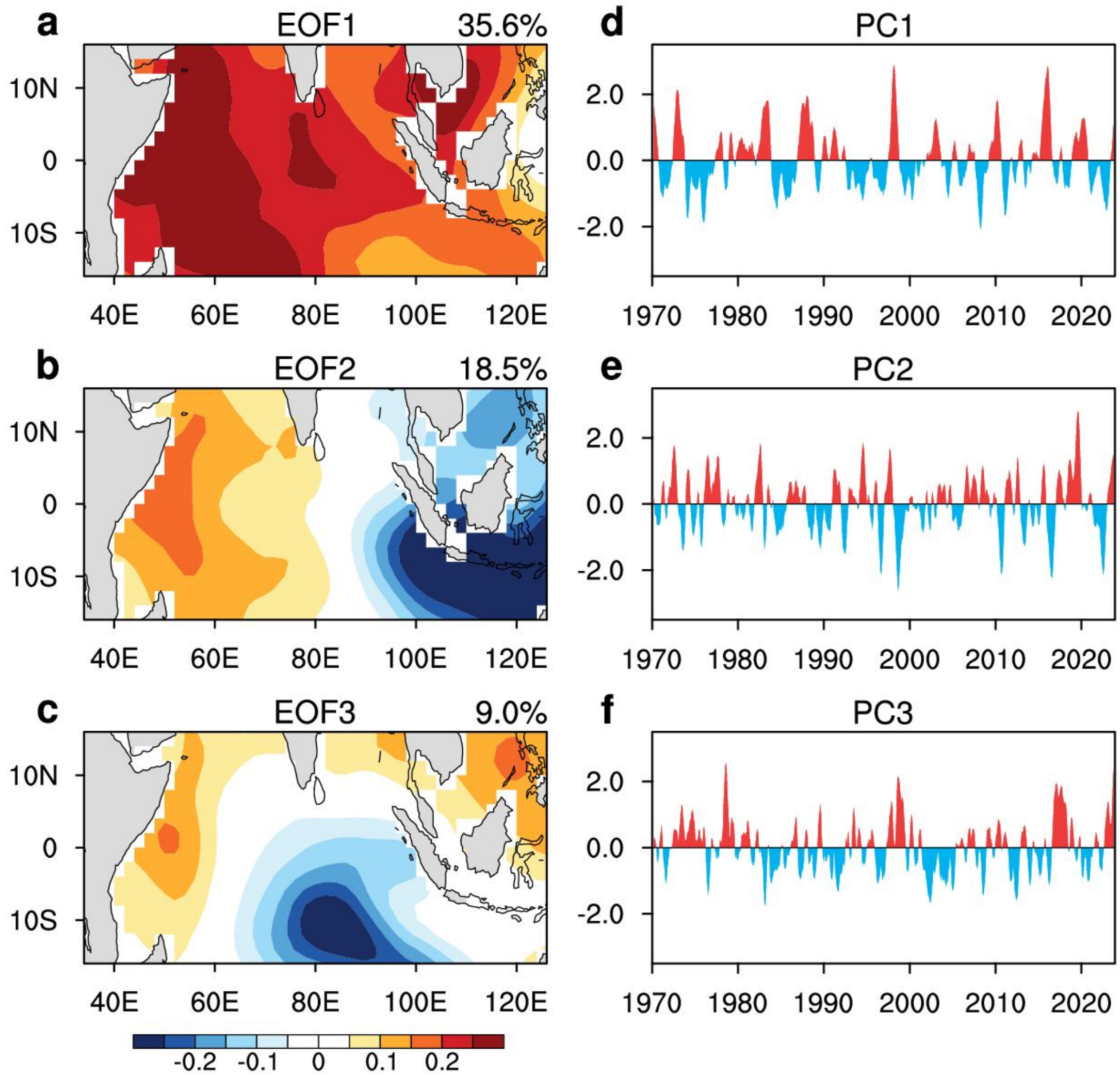

Figure S7. Leading EOF modes of SST anomalies in the tropical Indian Ocean. EOF analysis was performed over 34°E–126°E, 16°S–16°N for 1970–2023. (a–c) Spatial patterns of the first three EOF modes (°C, shading), with explained variance (%) indicated at the top right of each panel. (d–f) Time series of the corresponding PCs, smoothed with a 5-month running mean.

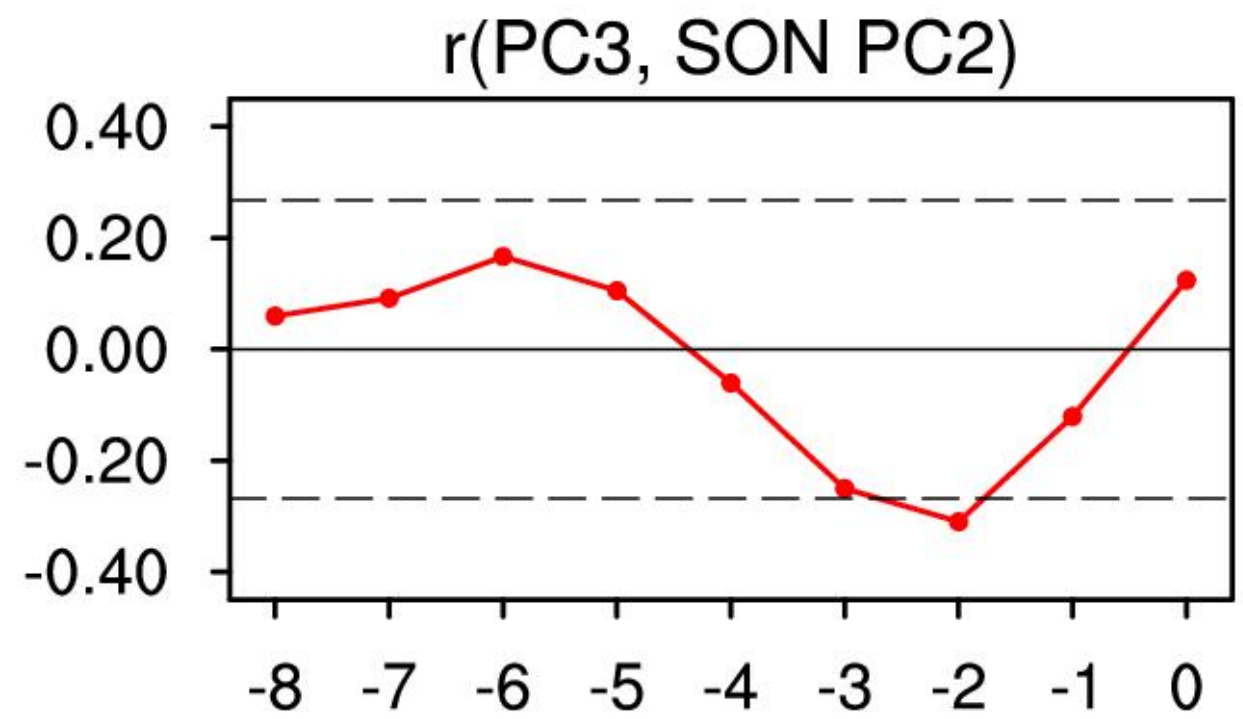

Figure S8. Lead correlations between seasonal-mean PC3 and SON PC2. The x-axis represents the lead time of PC3 relative to SON PC2 (e.g., a value of −1 indicates ASO PC3 preceding SON PC2). A significant correlation is found between JAS PC3 and SON PC2.

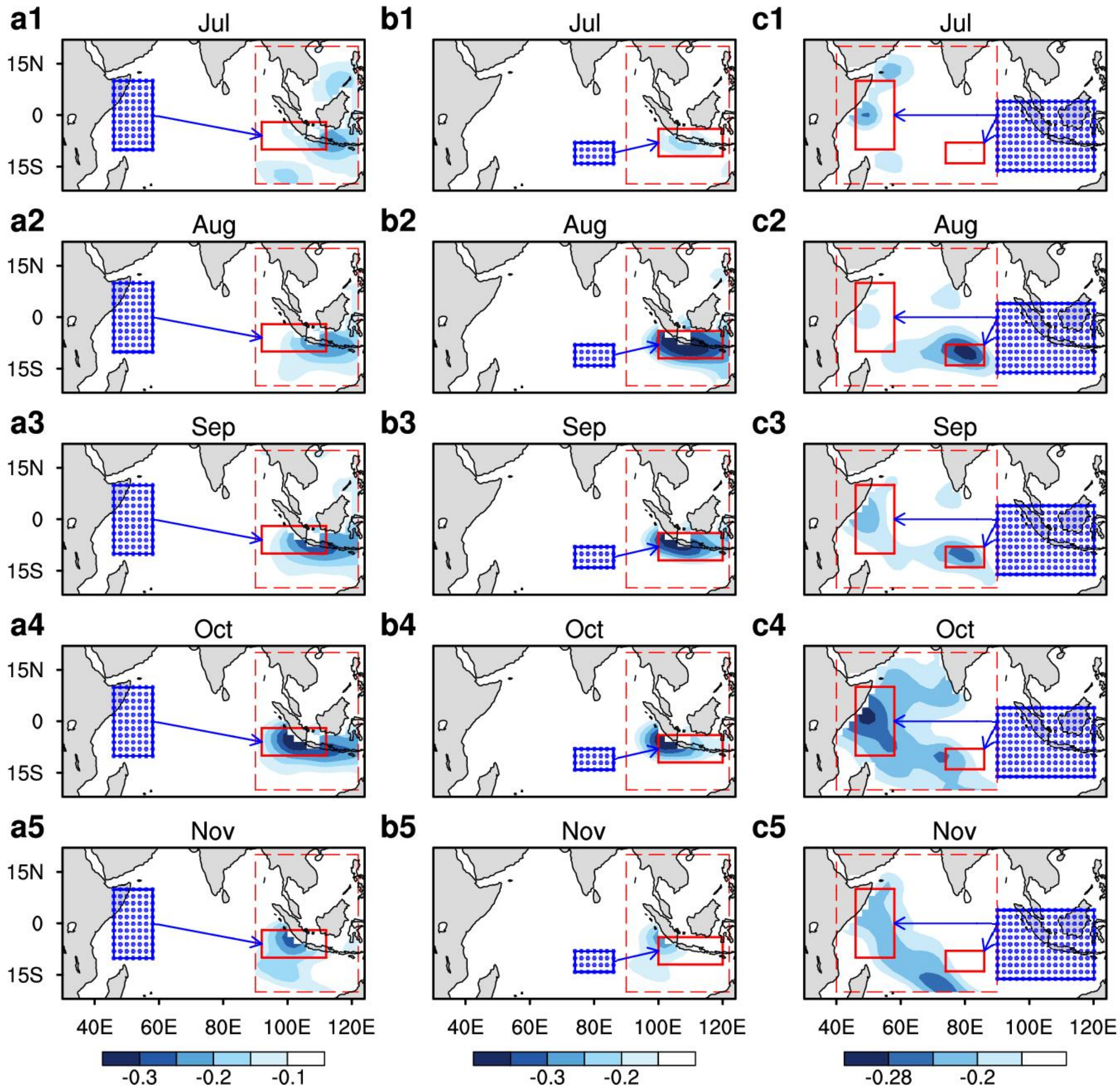

Figure S9. Identification of the two IOD types using the maximum negative response analysis method. (a1–a5) Maximum negative response (see Methods) of the eastern Indian Ocean SST (90°E–122°E, 20°S–20°N) to the western center (46°E–58°E, 10°S–10°N). (b1–b5) As in (a1–a5), but for the response of the eastern Indian Ocean SST to the central center (74°E–86°E, 14°S–8°S). (c1–c5) As in (a1–a5), but for the response of the western Indian Ocean SST (40°E–90°E, 20°S–20°N) to the broad eastern Indian Ocean region (90°E–130°E, 20°S–20°N). Blue boxes indicate the reference regions, red

dashed boxes indicate the target regions, and red solid boxes indicate the regions of maximum response used to define the two IOD types.

In October, as shown in (a4) and (c4), the strongest responses between the western center and eastern Indian Ocean region 1 (92°E–112°E, 10°S–2°S) form a dipole structure. In August, as shown in (b2) and (c2), the strongest responses between the central center and eastern Indian Ocean region 2 (100°E–120°E, 12°S–4°S) form another dipole structure. These two dipole structures can be defined as the W-IOD and the C-IOD, respectively, which is consistent with the IOD types defined in the main text using the EOF-based method.

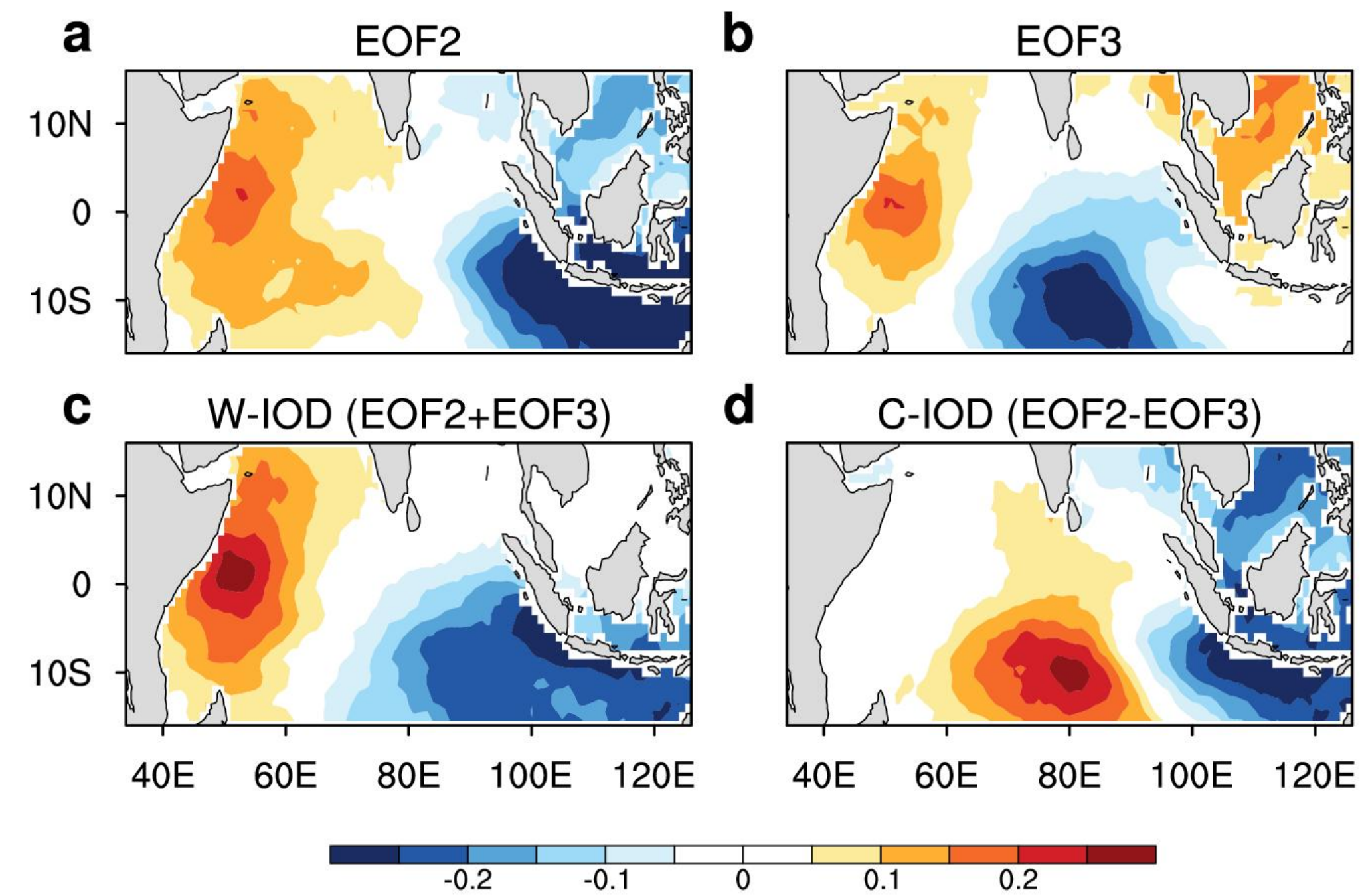

Figure S10. Reconstruction of the two IOD types using the EOF combinations based on the OISSTv2 dataset. EOF analysis was performed over 34°E–126°E, 16°S–16°N during 1982–2023. (a) EOF2, (b) EOF3, (c) $(\mathrm{EOF2} + \mathrm{EOF3})/\sqrt{2}$, and (d) $(\mathrm{EOF2} - \mathrm{EOF3})/\sqrt{2}$ (°C, shading). The results are consistent with those obtained from ERSSTv5.

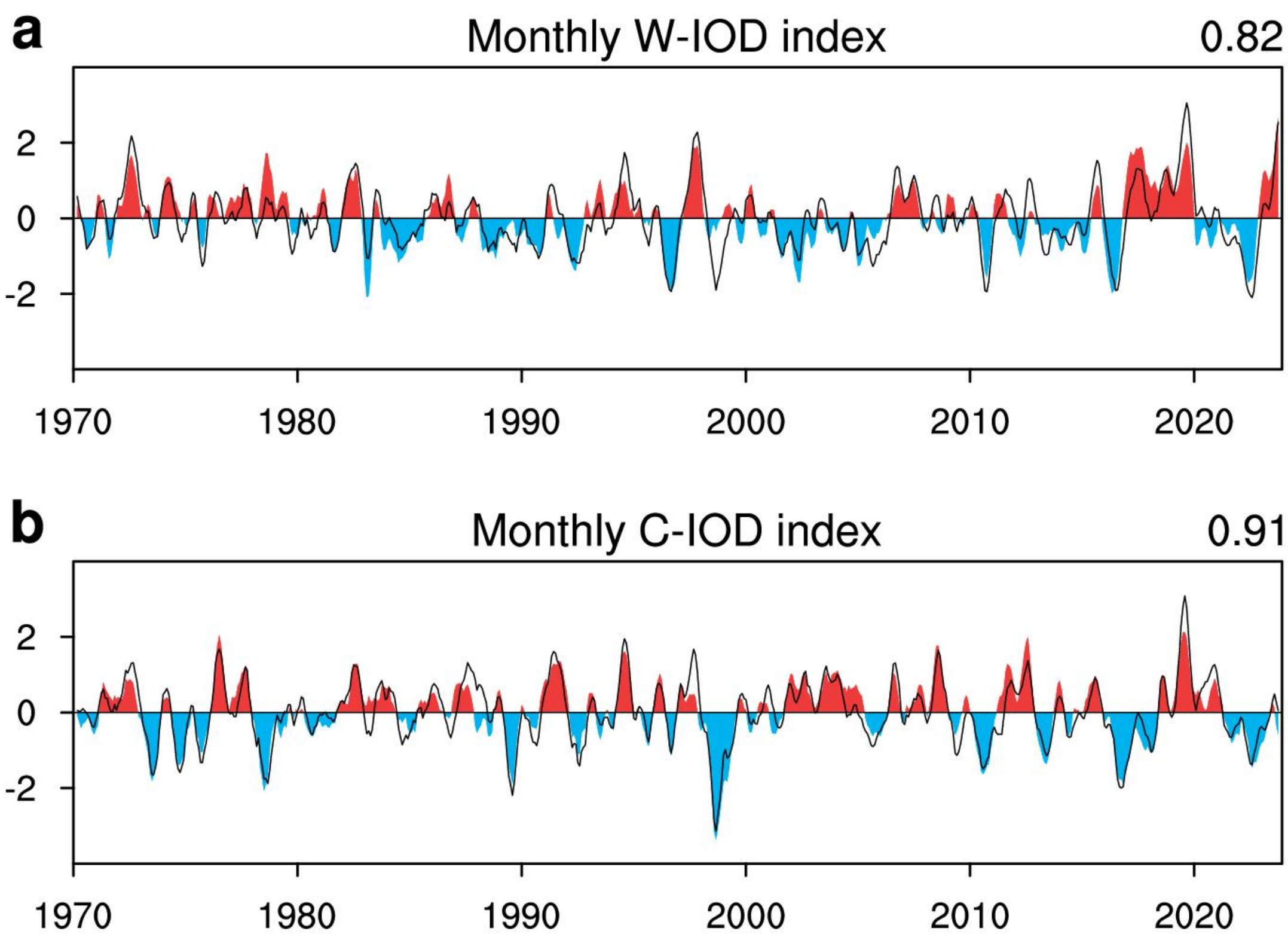

Figure S11. The W-IOD and C-IOD indices during 1970–2023, smoothed with a 5-month running mean. Two types of IOD indices are calculated from PC combinations (shaded curves) or from SST anomaly differences between the corresponding regions (black lines). The correlation between the two sets of indices is shown at the top right.

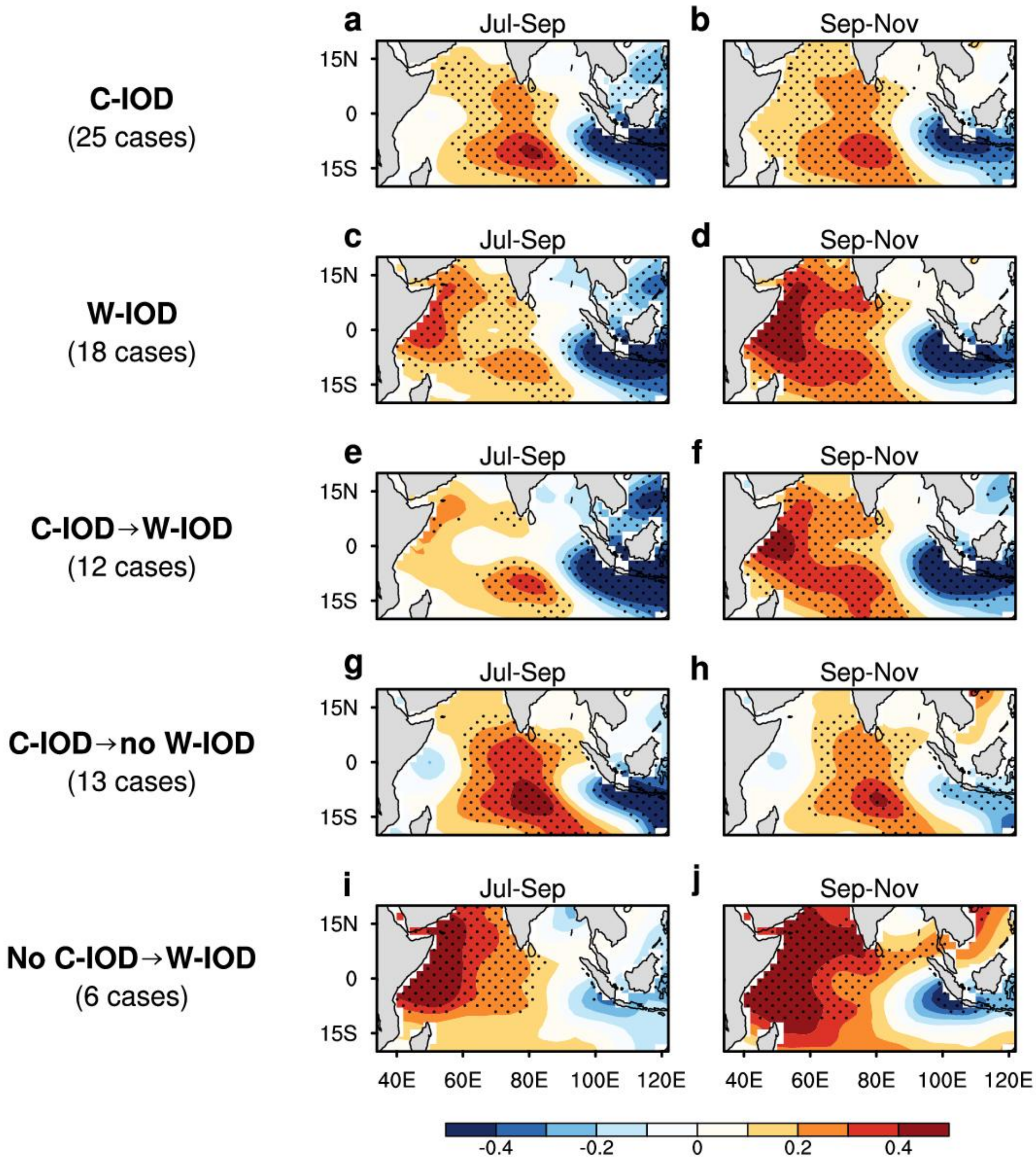

Figure S12. Evolution of SST anomalies (°C, shading) for C-IOD and W-IOD events under different phase relationships. (a and b) All C-IOD events. (c and d) All W-IOD events. (e and f) C-IOD events followed by a W-IOD. (g and h) C-IOD events not followed by a W-IOD. (i and j) W-IOD events not preceded by a C-IOD. Shading represents composited SST anomalies for both positive and negative phase years, with

negative-phase years multiplied by −1 to facilitate a unified comparison. Stippling indicates significance at the 95% level.

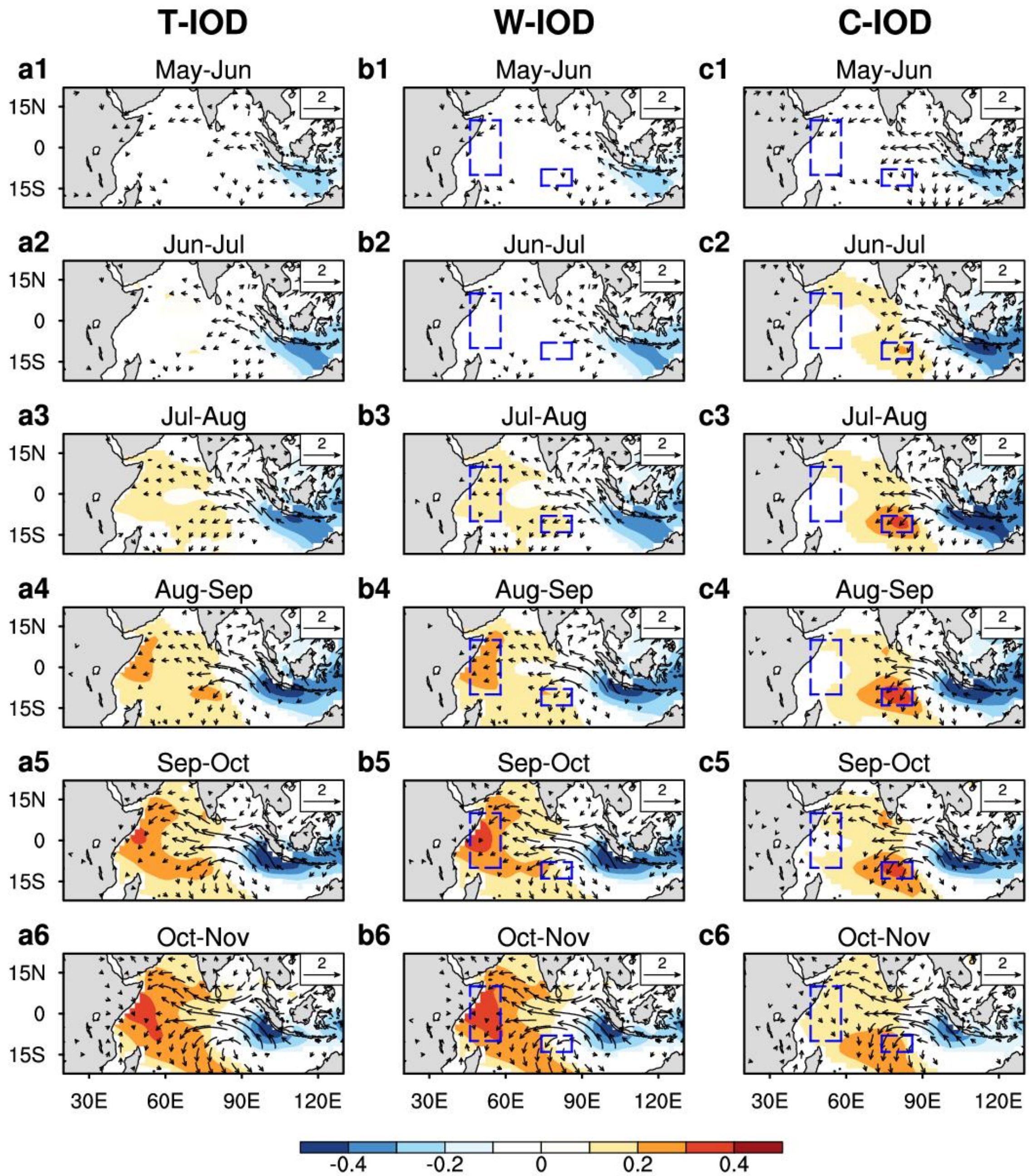

Figure S13. Evolution of bimonthly SST anomalies (°C, shading) and near-surface (σ = 0.995) wind anomalies (m s^{-1}, vectors) regressed onto the three standardized IOD indices during 1970–2023. Panels show regressions onto: autumn T-IOD index (a1–a6); autumn W-IOD index (b1–b6); summer C-IOD index (c1–c6). Only anomalies significant

at the 95% confidence level are shown. Blue dashed boxes indicate the western and central centers.

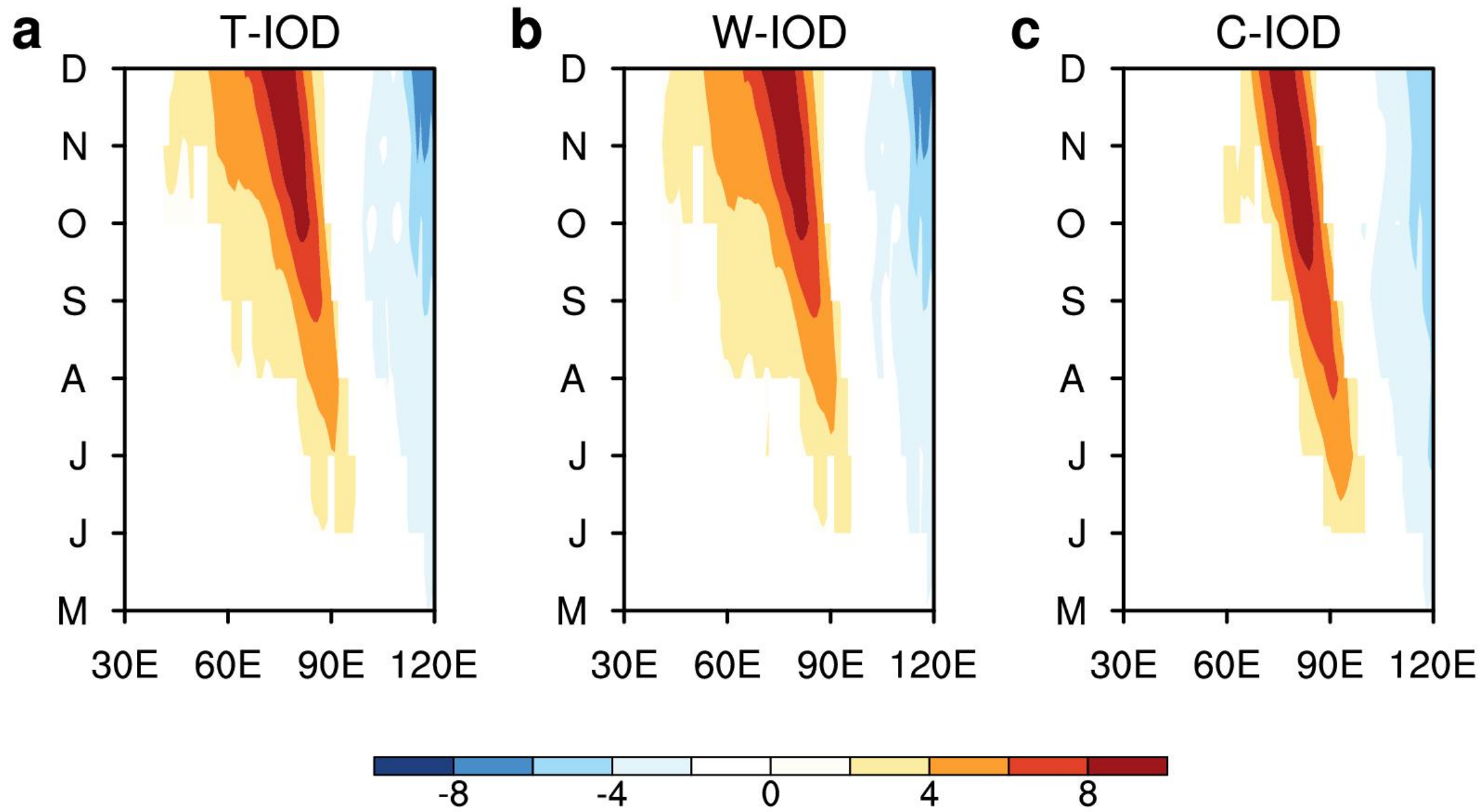

Figure S14. Longitude–time Hovmöller diagrams of SSH anomalies (cm, shading) at 10.5°S regressed onto the standardized IOD indices during 1970–2023. Panels show regressions onto: autumn T-IOD index (a); autumn W-IOD index (b); summer C-IOD index (c). Only anomalies significant at the 95% confidence level are shown.

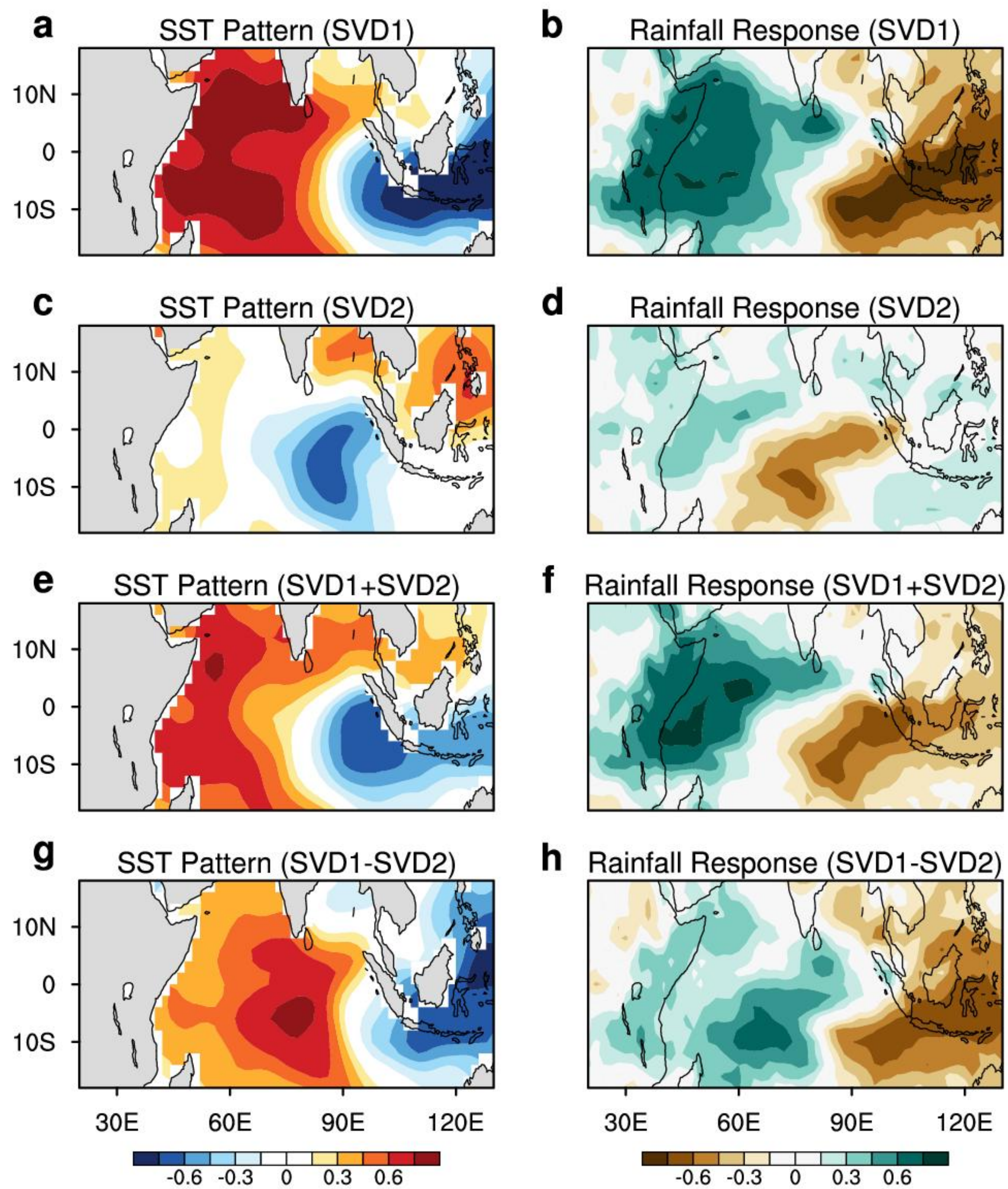

Figure S15. Coupled modes of SON SST and rainfall based on SVD analysis over the region 18°S–18°N, 20°E–130°E. (a and b) Homogeneous mode of SST anomalies (°C, shading) and heterogeneous mode of rainfall anomalies (mm, shading) derived from SVD1, respectively. (c and d) As in (a and b), but for SVD2. (e and f) As in (a and b), but

for the combined mode (SVD1+SVD2)/$\sqrt{2}$. (g and h) As in (a and b), but for the combined mode (SVD1-SVD2)/$\sqrt{2}$. The linear combination of the SVD modes in (e–h) demonstrates the distinct relationships between the two IOD patterns and East African rainfall.

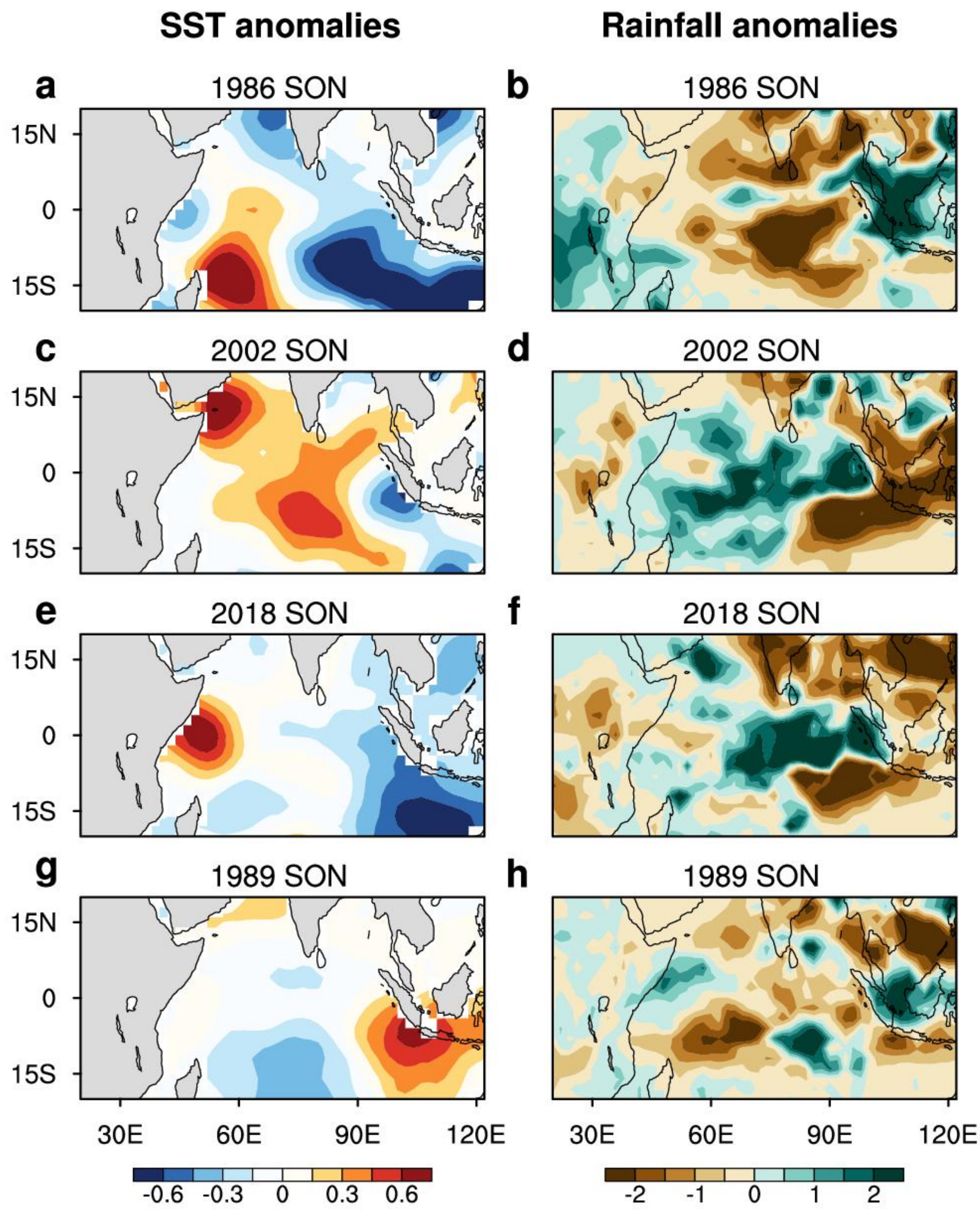

Figure S16. Boreal autumn SST anomalies (°C, left) and rainfall anomalies (mm, right) during four T-IOD events (1986, 1989, 2002, and 2018) that exhibited near-normal rainfall. The years 1986, 1989, and 2002 are classified as T-IOD events but are not W-

IOD events. The year 2018 is unusual, with strong negative SST anomalies and suppressed convection northwest of Australia. Abbreviation: SON, September–November.

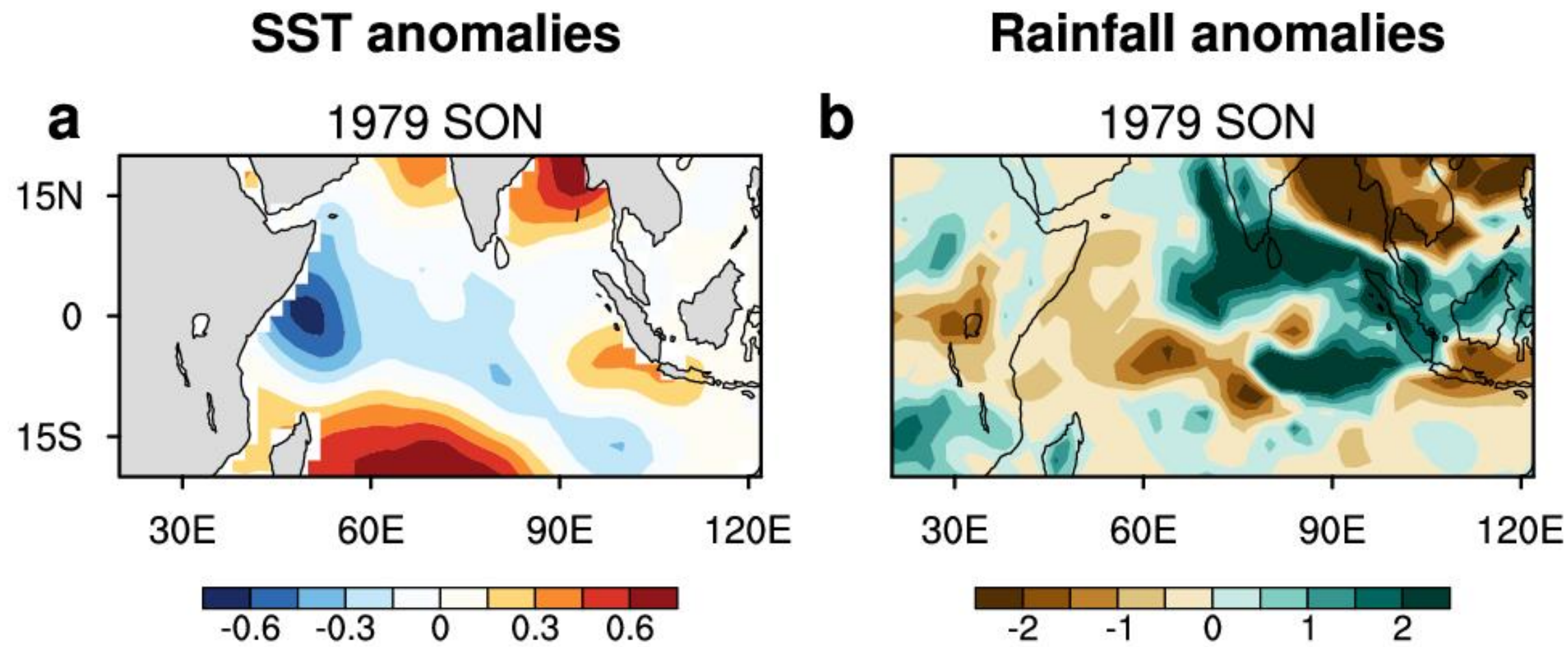

Figure S17. Boreal autumn SST anomalies (°C, left) and rainfall anomalies (mm, right) for 1979. This year represents an additional matched case, characterized by a negative W-IOD event associated with reduced East African rainfall, which was not identified by the T-IOD. Abbreviation: SON, September–November.

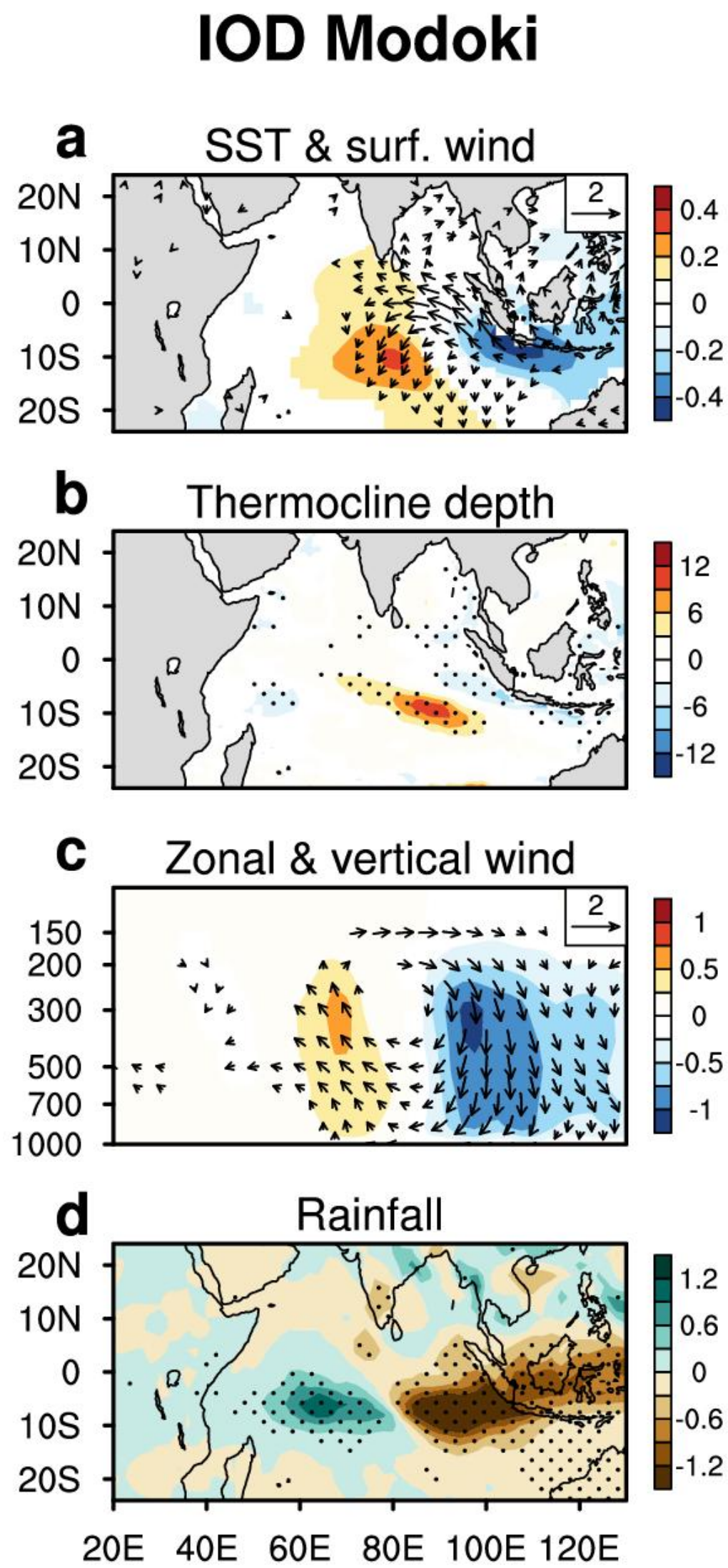

Figure S18. As in Fig. 3, but showing regression patterns onto the IOD Modoki index, which essentially mirror those of the C-IOD but with weaker strength. Here, the IOD Modoki index is calculated based on SST anomalies in three regions: IOD Modoki index = SSTA_central – 0.5 × SSTA_western – 0.5 × SSTA_eastern. The central box is 65°–85°E, 15°S–0°; the western box is 40°–55°E, 10°S–15°N; and the eastern box is 90°–110°E, 10°S–0°. These regions were originally introduced by Endo and Tozuka as classification criteria for IOD and IOD Modoki events [22].

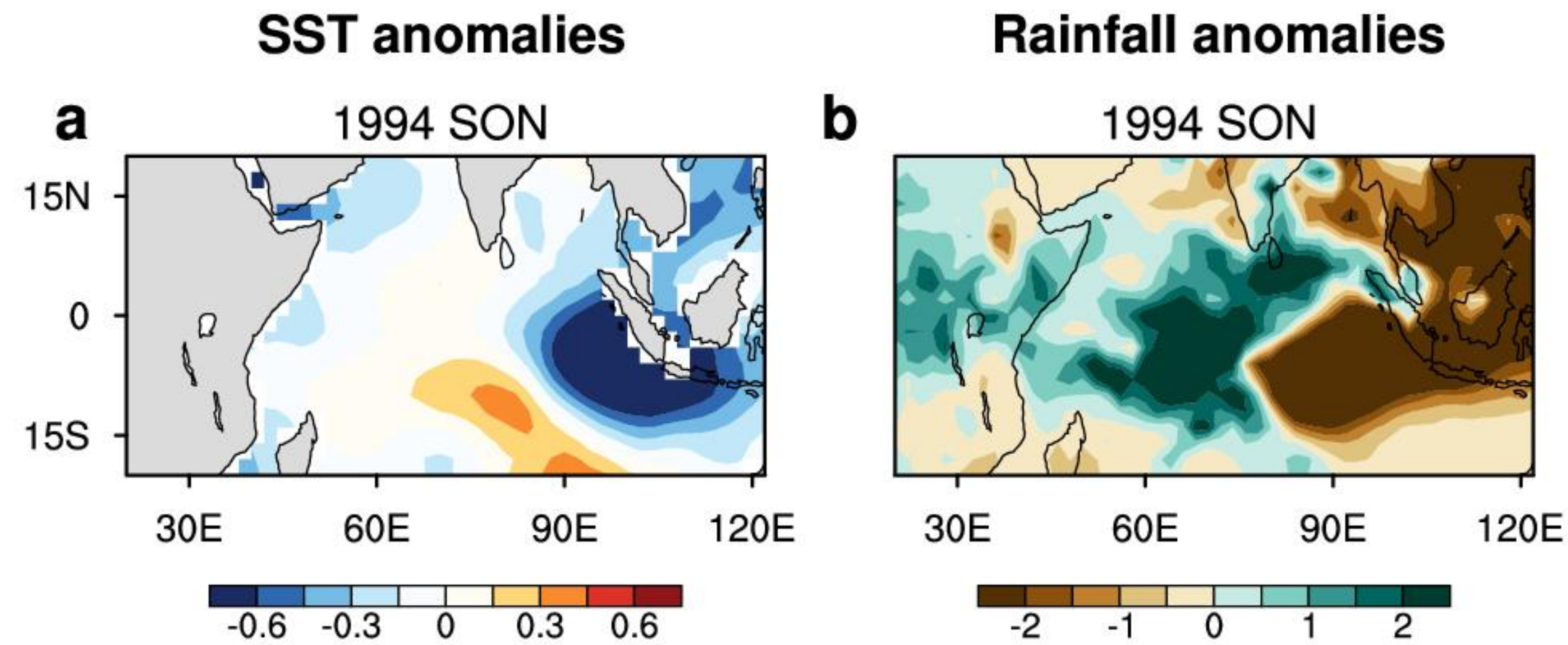

Figure S19. Boreal autumn SST anomalies (°C, left) and rainfall anomalies (mm, right) for 1994. Enhanced East African rainfall in this year can not be explained by the IOD Modoki, but rather by a positive W-IOD event, characterized by a weak cold SST anomaly in the western center and a strong cold SST anomaly in the eastern Indian Ocean.

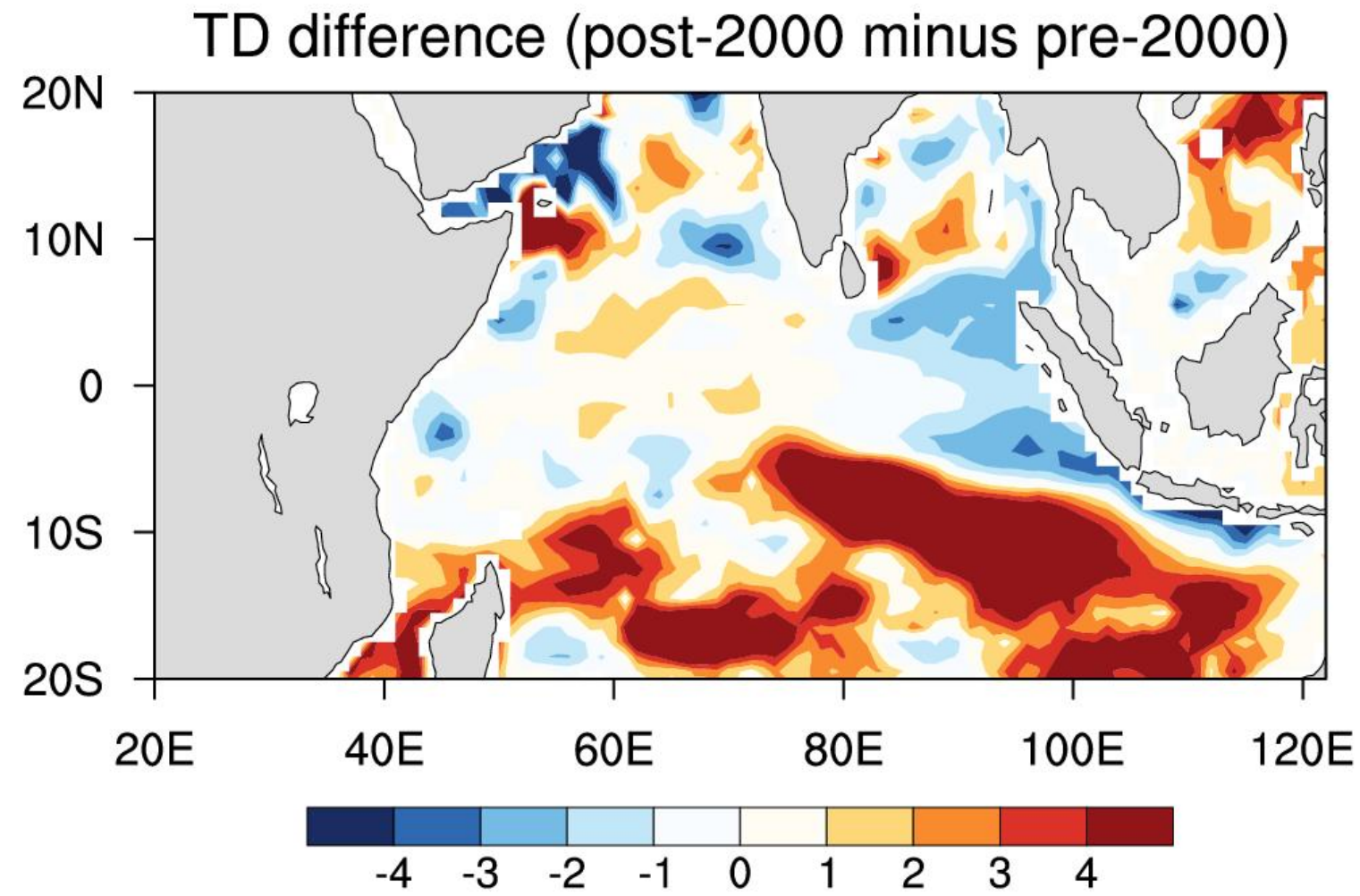

Figure S20. Change in July–November mean thermocline depth (m, shading) between 2000–2023 and 1970–1999. Abbreviation: TD, thermocline depth.

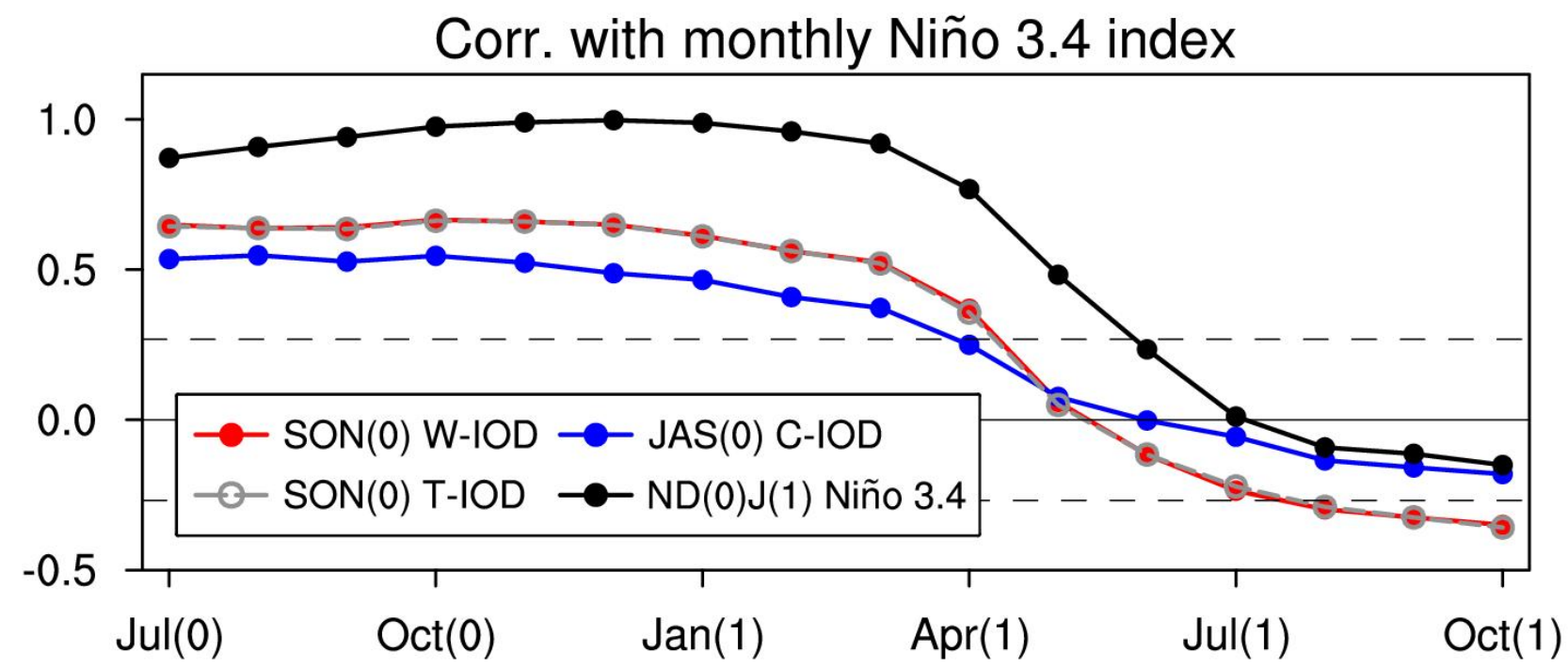

Fig. S21. Lead–lag correlations between the SON(0) W-IOD, SON(0) T-IOD, JAS(0) C-IOD, and ND(0)J(1) Niño-3.4 indices and monthly Niño-3.4 index. Here, (0) and (1) denote the same year and the following year, respectively. Red, gray dashed, blue, and black lines denote the lead–lag correlations for the W-IOD, T-IOD, C-IOD, and Niño-3.4 indices, respectively. Horizontal dashed lines indicate the 95% confidence level (±0.27). The W-IOD, similar to the T-IOD, is characterized by a pronounced sign reversal in its correlation with ENSO in the following year, whereas such a feature is absent for the C-IOD. The physical mechanisms underlying this contrast remain to be explored.

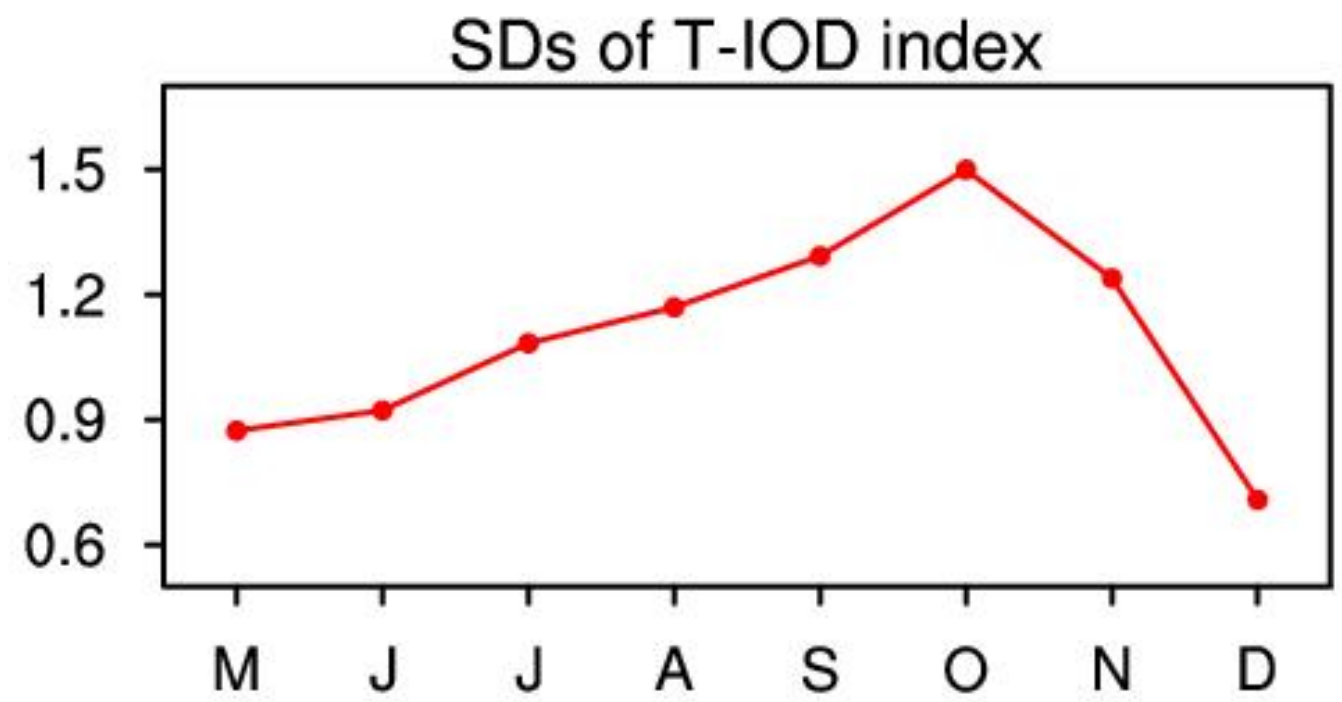

Figure S22. Standard deviations of the monthly T-IOD index for 1970–2023. The T-IOD index, defined as the SST anomaly difference between the tropical western (50°E–70°E, 10°S–10°N) and eastern (90°E–110°E, 10°S–0°) Indian Ocean [8], exhibits a strong monotonic increase prior to October. Abbreviation: SDs, standard deviations.

Tables

Table S1. Summary of existing and newly identified IOD classifications: definitions, spatial structures, indices, and event identification.

<table>
<tr><th colspan="2">IOD classifications</th><th>Description</th></tr>
<tr><td colspan="2" rowspan="4">T-IOD [8]</td><td>West–east dipole pattern, peaking in boreal autumn (SON).</td></tr>
<tr><td>Index = SSTA_western−SSTA_eastern, where western (50°E–70°E, 10°S–10°N) and eastern (90°E–110°E, 10°S–0°).</td></tr>
<tr><td>Based on a ±0.8σ threshold: 11 positive (1972, 1982, 1986, 1994, 1997, 2002, 2006, 2015, 2018, 2019, 2023) and 9 negative (1975, 1989, 1990, 1996, 1998, 2005, 2010, 2016, 2022).</td></tr>
<tr><td>Caveats: primarily derived from a leading EOF mode and may not fully capture dominant SST variability centers.</td></tr>
<tr><td rowspan="8">IOD Modoki and T-IOD [22]</td><td rowspan="4">IOD Modoki</td><td>Tripole pattern, peaking in boreal summer (JAS).</td></tr>
<tr><td>No explicit index defined; classification based on SST anomalies in western (40°E–55°E, 10°S–15°N), central (65°E–85°E, 15°S–0°), and eastern (90°E–110°E, 10°S–0°).</td></tr>
<tr><td>Based on central–eastern (±1.0σ) and central–western (±0.5σ) SST differences: 8 positive (1972, 1976, 1991, 1994, 2002, 2003, 2008, 2011) and 7 negative (1974, 1989, 1990, 1992, 1995, 2009, 2016).</td></tr>
<tr><td>Caveats: derived from a single event (the 1994 case); only a classification method was provided.</td></tr>
<tr><td rowspan="4">T-IOD (termed the canonical IOD in [22])</td><td>West–east dipole pattern, peaking in boreal autumn (SON).</td></tr>
<tr><td>Defined as the remaining events after excluding IOD Modoki events.</td></tr>
<tr><td>5 positive (1997, 2006, 2012, 2019, 2023) and 5 negative (1975, 1998, 2005, 2010, 2022).</td></tr>
<tr><td>Caveats: dependent on IOD Modoki removal; sensitive to classification scheme.</td></tr>
</table>

Indian Ocean tripole and T-IOD [24]	Indian Ocean tripole	Tripole pattern; peaking in JAS, defined as JJA in [24] to distinguish it from T-IOD.<br><br>Index = SSTA_central−0.5×SSTA_western−0.5×SSTA_eastern, where western (comprising 45°E–60°E, 5°S–20°N; 60°E–70°E, 10°N–20°N), central (65°E–85°E, 20°S–5°S) and eastern (90°E–110°E, 10°S–0°).<br><br>Based on a ±1.0σ threshold and a prescribed tripole structure requirement: 6 positive (1976, 1986, 1991, 1994, 2002, 2008) and 5 negative (1973, 1992, 1998, 2009, 2010).<br><br>Caveats: primarily derived from a leading EOF mode, and requires removal of PC2 in index calculation.
	T-IOD (termed the IOD in [24])	West–east dipole pattern, peaking in boreal autumn (SON).<br><br>Index = SSTA_western−SSTA_eastern, where western (45°E–60°E, 5°S–20°N) and eastern (90°E–110°E, 10°S–0°).<br><br>Based on a ±1.0σ threshold: 8 positive (1972, 1994, 1997, 2006, 2015, 2018, 2019, 2023) and 7 negative (1975, 1990, 1996, 1998, 2010, 2016, 2022).<br><br>Caveats: regarded as T-IOD, but with a westward-shifted western pole.
W-IOD and C-IOD (this study)	W-IOD	West–east dipole pattern, peaking in boreal autumn (SON).<br><br>Index = SSTA_western−SSTA_eastern, where western (46°E–58°E, 10°S–10°N) and eastern (92°E–112°E, 10°S–2°S).<br><br>Based on a ±0.8σ threshold: 9 positive (1972, 1982, 1994, 1997, 2006, 2015, 2018, 2019, 2023) and 9 negative (1975, 1979, 1990, 1996, 1998, 2005, 2010, 2016, 2022).<br><br>Contributions: based on multiple approaches, providing improved representation of SST variability.

C-IOD	Central–eastern dipole pattern, peaking in boreal summer (JAS).<br><br>Index = SSTA_central−SSTA_eastern, where central (74°E–86°E, 14°S–8°S) and eastern (100°E–120°E, 12°S–4°S).<br><br>Based on a ±0.8σ threshold: 14 positive-phase years (1976, 1977, 1982, 1983, 1987, 1991, 1994, 1997, 2003, 2006, 2008, 2012, 2018, 2019) and 11 negative-phase years (1973, 1974, 1975, 1978, 1989, 1990, 1992, 1998, 2010, 2016, 2022).<br><br>Contributions: based on multiple approaches, providing improved representation of SST variability.

Note: The listed years are identified for 1970–2023 using ERSSTv5, based on definitions in the respective studies. Abbreviation: JJA, June-August; JAS, July–September; SON, September–November; SSTA, SST anomalies.

Table S2. 21-year running mean frequency of T-IOD, W-IOD, and C-IOD events, averaged for pre- and post-2000 periods.

IOD Type	Phase	Averaged period	21-year running mean frequency (events/decade)	Difference (Post-2000 − Pre-2000) (events/decade)
T-IOD	Positive	Pre-2000	1.8	–
		Post-2000	2.1	0.3
	Negative	Pre-2000	1.8	–
		Post-2000	1.9	0.0
W-IOD	Positive	Pre-2000	1.2	–
		Post-2000	1.6	0.5
	Negative	Pre-2000	1.6	–
		Post-2000	1.9	0.3
C-IOD	Positive	Pre-2000	2.9	–
		Post-2000	2.7	-0.2
	Negative	Pre-2000	2.2	–
		Post-2000	1.3	-1.0

Note: The terms “pre-2000” and “post-2000” denote the 21-year running mean periods centered on 1980–1999 and 2000–2013, respectively.
